# DNA replication and repair kinetics of Alu, LINE-1 and satellite III genomic repetitive elements

**DOI:** 10.1186/s13072-018-0226-9

**Published:** 2018-10-23

**Authors:** Francesco Natale, Annina Scholl, Alexander Rapp, Wei Yu, Cathia Rausch, M. Cristina Cardoso

**Affiliations:** 1 Department of Biology, Technische Universität Darmstadt, 64287 Darmstadt, Germany; 2 Present Address: Biology Unit, IRBM Science Park S. p. A., 80131 Naples, Italy; 3 Present Address: G5 Lymphocyte Development and Oncogenesis, Immunology Department, Pasteur Institute, 75724 Paris Cedex 15, France

**Keywords:** Alu, ChIP-Seq, DNA repair, DNA repetitive elements, DNA replication, Genome-wide analysis, Immuno-FISH, LINE, Phosphorylated H2AX, Satellites

## Abstract

**Background:**

Preservation of genome integrity by complete, error-free DNA duplication prior to cell division and by correct DNA damage repair is paramount for the development and maintenance of an organism. This holds true not only for protein-encoding genes, but also it applies to repetitive DNA elements, which make up more than half of the human genome. Here, we focused on the replication and repair kinetics of interspersed and tandem repetitive DNA elements.

**Results:**

We integrated genomic population level data with a single cell immunofluorescence in situ hybridization approach to simultaneously label replication/repair and repetitive DNA elements. We found that: (1) the euchromatic Alu element was replicated during early S-phase; (2) LINE-1, which is associated with AT-rich genomic regions, was replicated throughout S-phase, with the majority being replicated according to their particular histone marks; (3) satellite III, which constitutes pericentromeric heterochromatin, was replicated exclusively during the mid-to-late S-phase. As for the DNA double-strand break repair process, we observed that Alu elements followed the global genome repair kinetics, while LINE-1 elements repaired at a slower rate. Finally, satellite III repeats were repaired at later time points.

**Conclusions:**

We conclude that the histone modifications in the specific repeat element predominantly determine its replication and repair timing. Thus, Alu elements, which are characterized by euchromatic chromatin features, are repaired and replicated the earliest, followed by LINE-1 elements, including more variegated eu/heterochromatic features and, lastly, satellite tandem repeats, which are homogeneously characterized by heterochromatic features and extend over megabase-long genomic regions. Altogether, this work reemphasizes the need for complementary approaches to achieve an integrated and comprehensive investigation of genomic processes.

**Electronic supplementary material:**

The online version of this article (10.1186/s13072-018-0226-9) contains supplementary material, which is available to authorized users.

## Background

Preservation of genome integrity by complete, error-free DNA duplication prior to cell division and by correct DNA damage repair is paramount for the development and maintenance of an organism. This holds true not only for the protein-encoding genes, but also applies for the repetitive DNA elements [1]. More than half of the human genome is made up of repetitive DNA elements. This fraction is remarkably large when compared to the ~ 1.2% protein coding DNA [2]. In mouse, the proportion is similar, with repetitive DNA elements and coding regions making up to 40% and 1.4% of the murine genome, respectively [3].

Long interspersed nuclear elements (LINEs), short interspersed nuclear elements (SINEs) and LTR retrotransposons are transposable DNA elements. These elements insert into new genomic locations by reverse transcription of an RNA intermediate. LINEs are found in all vertebrate species. The LINE-1 (L1) family of transposable elements represents the only group of autonomous non-LTR retrotransposons in the human genome [4]. Functional L1 elements encode a consensus sequence of about 6 kbp, including two open reading frames encoding for proteins that are necessary for the retrotransposition [5–8]. L1 retrotransposition requires transcription of L1 RNA, its transport to the cytoplasm, and translation of its two open reading frames. Both L1-encoded proteins (ORF1p and ORF2p) are thought to preferentially associate with their own encoding RNA and form a ribonucleoprotein complex, which is a proposed retrotransposition intermediate [9]. The latter must then access the nucleus, where the L1 endonuclease cleaves the genomic DNA at a degenerate consensus sequence. The resulting free 3′ hydroxyl residue is subsequently used by the L1 reverse transcriptase as a primer to copy the L1 sequence in situ. Such process is termed “target-primed reverse transcription” [10]. Finally, the resulting L1 cDNA is joined to the target DNA. L1 elements alone make up about 17% of the human genome, and they are preferentially found at AT-rich and gene poor regions, corresponding to G-bands and DAPI-bright bands of metaphase chromosomes [2, 11]. However, the vast majority (> 99%) of L1s are, on average, 1400 bp long and inactive because of point mutations, truncations and other rearrangements; it is estimated that the average diploid human genome contains about 100 retrotransposition-competent L1s [10].

Alu repetitive DNA elements are among the most abundant SINEs. They are about 280 bp long and are specific to primates. Similar SINEs can be found in other organisms, like B1 elements in rodents [12]. Alu elements do not encode proteins but contain a RNA polymerase III promoter [2], and it was demonstrated that they use L1-encoded proteins for their retrotransposition in trans [13, 14]. There exist more than 10^6^ Alu elements in the human genome covering approximately 11% of the genomic DNA, and they are preferentially distributed in gene-rich genomic regions corresponding to R-bands in metaphase chromosomes [11]. Therefore, based on their genomic distribution, L1 and Alu elements represent chromatin compartments with opposing features. Furthermore, L1 and, potentially, Alu activity represents a potential threat for the integrity and stability of the genome, in both dividing and nondividing cells. By direct insertion of the transposable element into or close to a gene, L1 might interfere with gene activity, disrupting exons or influencing splicing [4]. Furthermore, because of their abundance, their sequences may be used in homologous recombination (HR) in a non-allelic fashion, leading to insertions or deletions in the damaged region [15, 16]. Indeed, insertions of L1 elements have been reported in tumor suppressor genes in several cancer types [17–19].

Satellite DNA elements consist of very large arrays of tandemly repeating, non-coding DNA. They are the main component of functional centromeres and form the main component of constitutive heterochromatin. Human satellite III and murine major satellite are examples of pericentromeric heterochromatin, while human alpha satellite and mouse minor satellite can be found in centromeres [20–24]. Mouse major satellite reaches up to 8 Mbp and is made up of 234 bp-long AT-rich units. It is found in pericentromeric regions of all chromosomes, except for the Y chromosome. In interphase nuclei, major satellite DNA can be found at bright DAPI-stained DNA regions. The latter consist of clusters of heterochromatic regions from several chromosomes and are known as “chromocenters” [3, 20]. Human satellite III consists of a 5-bp-long unit and its presence was shown in seven autosomes (chromosomes 1, 9, 13, 14, 15, 21 and 22) and the Y chromosome [21]. Despite their epigenetic state, pericentromeric satellites (e.g., satellite III) have been shown to be transcriptionally active in response of various stressors (UV-C, genotoxic chemicals, osmotic imbalance, oxidative stress and hypoxia). Transcription of these elements not only can stabilize pericentromeric regions, but also it can promote recovery from stress by activating alternative splicing and, thus, modulating critical stress response genes [25–27]. Finally, a large number of repetitive DNA elements can exist in at least two conformations: a right-handed B form (the most abundant) with canonical Watson–Crick base pairing and non-B conformations, possibly transiently formed at specific sequence motifs. The latter may arise from supercoil density, partly generated by transcription or protein binding, and are involved in genome susceptibility to DNA damage [28].

Overall, the DNA duplication and repair of repetitive DNA elements before cell division are paramount to genome integrity. However, the spatio-temporal organization of DNA duplication and repair of repetitive elements is yet to be fully elucidated. In this study, we investigated the DNA replication timing and DNA double-strand break repair kinetics of different repetitive DNA elements. We integrated publicly available genomic data (ChIP-Seq, Repli-Seq and other repetitive and non-B DNA sequences) with a combined immunofluorescence in situ hybridization (FISH) analysis to visualize DNA replication or DNA damage response (DDR) sites in the context of repetitive DNA elements. Our results reemphasize the need for complementary approaches to achieve an integrated and comprehensive investigation of any genomic process.

## Results

### Genome-wide replication timing of repetitive DNA elements correlates with GC content, gene density and chromatin state

First, we asked whether the replication timing of repetitive DNA elements may depend on the genomic distribution and, thus, the chromatin state of such elements. To characterize such relation, we retrieved publicly available genomic data of 12 different human repetitive elements, covering all different types: direct, inverted, mirror, tandem (microsatellite/SSRs), low complexity (AT and GC) and interspersed elements. The latter were dissected further into SINEs (Alu and MIR), LINEs (L1 and L2) and LTR/DNA transposons (MER) without further subdivision into subfamilies (Table 1; [29, 30]). These classifications utilize the reference genome assembly and are not ploidy-adjusted.

**Table 1:** Overview of human repetitive and non-B DNA elements

DNA sequence element	Type of sequence	Data source [29, 30]
Alu sequence	Short interspersed nuclear element	RepeatMasker
MIR	Short interspersed nuclear element	RepeatMasker
LINE1	Long interspersed nuclear element	RepeatMasker
LINE2	Long interspersed nuclear element	RepeatMasker
MER	LTRs/DNA transposons	RepeatMasker
AT low complexity repeats		RepeatMasker
GC low complexity repeats		RepeatMasker
Simple sequence repeats (SSRs)	Tandem repeats	RepeatMasker
G-Quadruplex forming repeats	non-B DNA structure	RepeatMasker
Z-DNA motif	non-B DNA structure	Non-B DataBase
Inverted repeats	Repetitive DNA element	Non-B DataBase
Cruciform motif	non-B DNA structure	Non-B DataBase
Direct repeats	Repetitive DNA element	Non-B DataBase
Slipped motif	non-B DNA structure	Non-B DataBase
Mirror repeats	Repetitive DNA element	Non-B DataBase
Triplex motif	non-B DNA structure	Non-B DataBase
A-Phased motif	non-B DNA structure	Non-B DataBase
Microsatellite	Tandem repeats	RepeatMasker

**Fig. 1:**
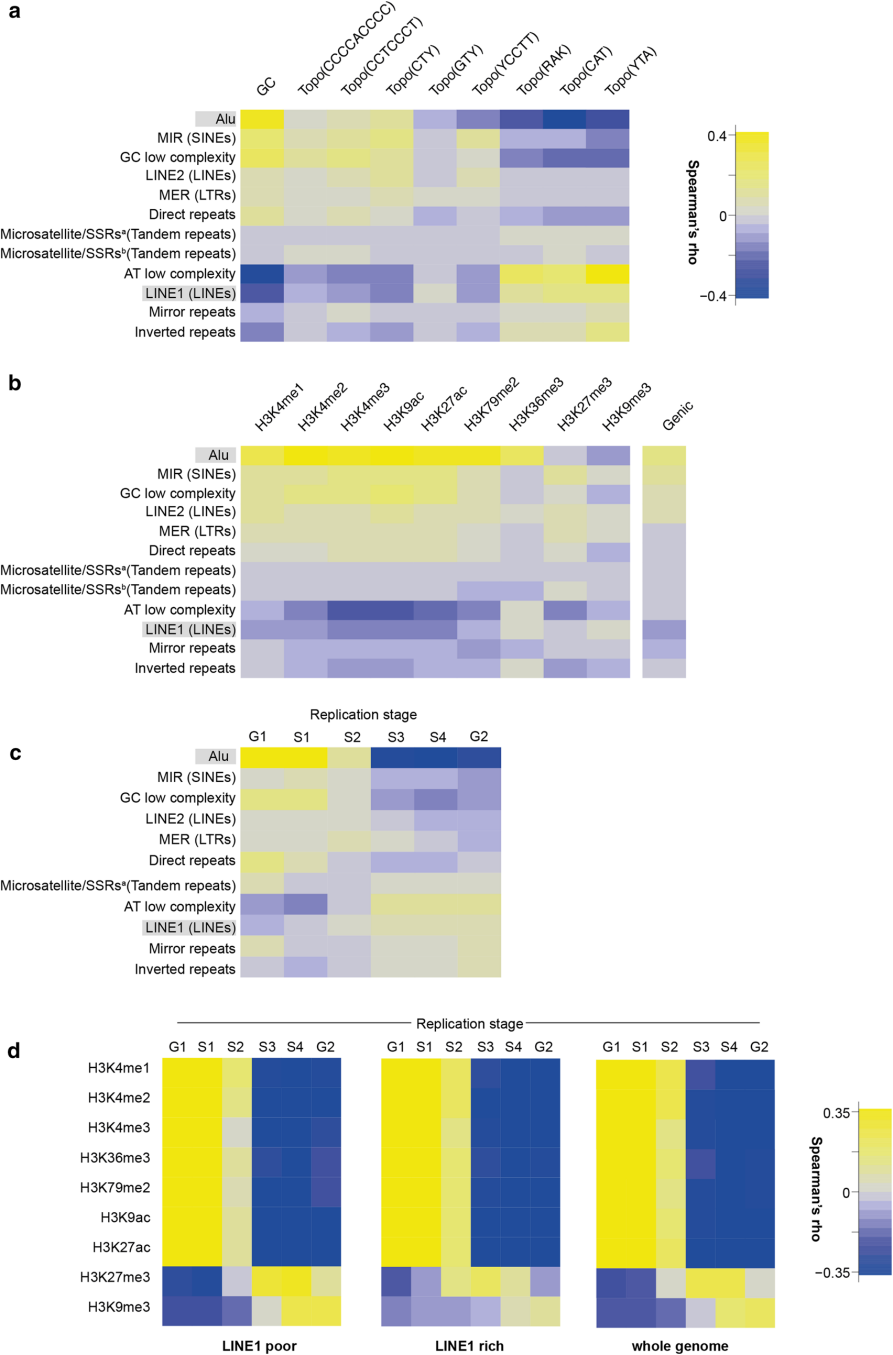
Genomic features of repetitive DNA elements. Spearman’s rho correlation matrix. The number of each repetitive DNA element copies, or the amount of a given genomic feature is counted in each 10 kb genomic interval. The correlation coefficient is calculated for comparison of repetitive DNA elements with GC content (**a**), histone modifications (from HeLa, ENCODE tier 2) or genic regions (**b**) and replication timing from Repli-Seq data (HeLa, ENCODE tier 2) [31] (**c**). Data are from > 290,000 genomic intervals. For each correlation, *P* < 2.2 × 10^−16^. In **a**, Topo(…): from left to right, topoisomerase I consensus sequences at decreasing GC content. Highlighted Alu and L1 repetitive elements are arbitrarily chosen to define chromatin compartments with opposing chromatin features, and are further investigated in FISH experiments. **d** Correlation matrix of histone modifications and replication timing in HeLa cells, for L1-rich (> 10 counts per genomic interval) L1-poor (> 1 count per genomic element) genomic regions

DNA sequence composition is one of the genomic features dictating the distribution of repetitive DNA elements in the genome. Hence, we started with computing the abundance of the 12 different repetitive DNA elements (i.e., their number) in 10 kbp genomic intervals, which is the genomic resolution we adopted in this study (example tracks are given in Additional file 1: Fig. S1). Then, we calculated the genome-wide Spearman’s rho correlation coefficient between each repetitive DNA element abundance and different GC (or AT) content. Alu, GC low complexity, MIR and, to a minor extent, direct repeats were positively correlated with GC content, whereas AT low complexity and L1 were negatively correlated to GC content (Fig. 1a, left column). Repetitive DNA elements showing positive correlation with GC content also showed a gradually decreasing correlation (or an anti-correlation) with decreasing GC content (Fig. 1a). The inverted case was observed for those repetitive DNA elements showing a negative correlation with GC content, instead. Little to no correlation with GC content was observed for microsatellites/SSRs and mirror repeats (Fig. 1a).

Next, we investigated the relation between the above-mentioned repetitive elements and diverse histone modifications retrieved from publicly available databases (HeLa S3 cells, [ENCODE tier 2]). We computed the genome-wide Spearman’s rho correlation coefficient between each DNA repetitive element and each given histone modification. Repetitive DNA elements scoring an (anti-)correlation with GC content also scored a strong (anti-)correlation with the majority of the histone modifications we tested. For example, Alu elements were abundant on genomic locations whose chromatin was decorated by typical active promoter (H3K4me3/2, H3K9ac, H3K27ac) or gene body (H3K36me3, H3K79me2) modifications. Conversely, Alu elements were scarce on genomic regions whose chromatin was marked by H3K9me3 (Fig. 1b). Overall, Alu abundance positively correlated with genic regions (Fig. 1b, right column). L1 elements showed the opposite behavior with an anti-correlation to most euchromatic marks and genic elements and a weak positive correlation to H3K9me3. This observation may have implications in the DDR and is discussed below.

Finally, we correlated the abundance of the above-mentioned repetitive DNA elements with the stages of the S-phase of the cell cycle obtained by publicly available Repli-Seq experiments from HeLa S3 cells (ENCODE, tier 2) [31]. The Spearman’s correlation coefficient computed between Repli-Seq data and the above-mentioned repetitive DNA elements indicated that Alu elements were abundant on chromatin regions whose DNA was duplicated in the early G1b and S1 stages, but poorly represented in those regions duplicated in the S3, S4 and G2 late stages (Fig. 1c). Interestingly, despite showing a negative correlation with transcription-permissive histone modifications, L1 showed little to no correlation to any S-phase replication substage with L1 DNA being duplicated throughout S-phase with only a slight increase in mid and late S-phase (Fig. 1c).

We then asked whether the chromatin landscape played a role in the DNA replication of L1 elements and, specifically, in genomic regions where L1 elements are abundant. To this end, we segmented the genome into L1-rich (containing more than 10 L1 elements per 10 kpb interval) and L1-poor (no L1 elements), and then we computed the correlation coefficients between histone marks and replication substages. All transcription-permissive histone modifications showed a positive correlation with early S-phase (G1b and S1), independent of the abundance of L1 elements (Fig. 1d). This indicates that chromatin regions decorated with transcription-permissive marks were replicated during the early stage of S-phase. For these regions, we observed a transition phase during the mid-stage of S-phase, wherein the positive correlation shifted toward a negative correlation (Fig. 1d, S2 and S3), the latter persisting through the late substages of S-phase (Fig. 1d, S3 and S4). Heterochromatin-associated histone modifications (H3K27me3 and H3K9me3) showed a marked difference between L1-poor and L1-rich genomic regions, instead. Specifically, the latter presented reduced correlations throughout the cell cycle, suggesting an independent DNA replication mechanism throughout S-phase, once DNA replication commenced. Genomic regions devoid of L1 elements presented a pattern similar to that we observed for the whole genome, with H3K27me3/H3K9me3-decorated chromatin being replicated in the late stage of the S-phase.

To test the generality of our observations, we analyzed two additional cell lines: the lymphoblastoid GM12878 cells and the hepatocellular carcinoma HepG2. Similar to our observations in HeLa cells, an identical temporal pattern was observed for transcription-permissive histone modifications (Additional file 1: Fig. S2). Heterochromatin-associated histone modifications presented marked cell-specific differences, instead. Specifically, HepG2 cells presented a late replication of H3K9me3-decorated chromatin (Additional file 1: Fig. S2c). The latter was replicated much earlier in GM12878 cells, instead (Additional file 1: Fig. S2b). Further, L1 abundance seemed to have a cell-specific impact on the correlation between heterochromatin-associated histone modifications and replication timing: while we observed a difference between L1-poor and L1-rich genomic regions in GM12878 cells, no difference was observed in HepG2 cells. For the former, it was the absence of L1 elements that led to a decrease in the correlation coefficient throughout the S-substages.

Taken together, these observations reveal that the histone modifications predominantly dictate the replication timing. Yet, the presence of L1 elements—and possibly their transcriptional context—locally perturbs the replication program.

### FISH-based replication timing assessment of interspersed and tandem repeats

Despite their high throughput and the high attainable read depth, it proves challenging to quantitatively assess highly repetitive DNA regions such as (peri-)centromeric chromatin by next-generation sequencing approaches. Sequences at the boundary of a given highly repetitive DNA region can be mapped by utilizing the non-repetitive (and, thus, mappable) adjacent sequences. However, peri- and centromeric regions, covering up to 15% of the genome, are harder to be probed by sequencing methods, especially for quantitative analyses. Therefore, to assess the DNA duplication prior to cell division of (peri-)centromeric chromatin, we established an immuno-FISH-based method. Together with (peri-)centromeric chromatin, we also probed Alu and L1 repetitive DNA elements, as they recapitulate chromatin compartments with opposing functional features (e.g., euchromatin versus heterochromatin) (Fig. 1b). Specifically, we investigated whether the repetitive DNA elements showed a temporal replication similar to that of the chromatin compartment they are mainly associated with. To address this question, we combined FISH, with probes for the repetitive DNA elements, with the detection of incorporated thymidine analogues to dissect the S-phase stages. Different chromatin types are replicated at different stages of the S-phase, which are identifiable by their spatial patterns [32]. Thus, we incubated unsynchronized HeLa (and C2C12) cells with 10 µM EdU for 15 min to label the replicating DNA, and then probed Alu, L1 and satellite III (and major satellite) elements with specific DNA probes.

First, we validated the specificity of the hybridization probes on mitotic chromosomes. As mentioned before, Alu elements are predominantly found in GC-rich regions and, thus, R-bands, whereas L1 are more abundant in GC-poor regions (or AT-rich). To counterstain the DNA, we employed DAPI, which preferentially binds to AT-rich DNA sequences. We simultaneously hybridized a biotin-labeled Alu probe and a digoxigenin labeled L1 probe on HeLa metaphases. Alu and L1 were indeed found to negatively correlate in color line profiles and Pearson’s correlation analysis showed anti-correlation of Alu and L1 with a ρ value of − 0.23 (Additional file 1: Fig. S3a). In addition, we probed human satellite III DNA, which forms constitutive heterochromatin with a probe specific for satellite III sequences found on chromosome 1. According to previous spectral karyotype analysis of HeLa cells [33], about four copies of satellite III per cell are expected. We observed more than four hybridization signals, indicating that other satellite III (or satellite II) sequences (most likely on chromosomes 9 and 16) were probed under our experimental conditions (Additional file 1: Fig. S3a). As a control, we also probed murine major satellite DNA in C2C12 myoblast cells. Each telocentric mouse chromosome possesses a major satellite DNA sequence, which was efficiently labeled by the probe (Additional file 1: Fig. S3a).

To investigate the replication timing of the above-mentioned repetitive elements in interphase, we measured the degree of colocalization by means of the *H* coefficient (*H*_coefficient_) as well as the Pearson’s correlation coefficient ($\rho$) [34]. The greater the *H*_coefficient_ is (> 1), the more the two signals colocalize. *H*_coefficient_ values lower than 1 indicates the two signals are randomly distributed. For example, L1 FISH signal (AT-rich) was more correlated with the DAPI signal than Alu FISH signal in interphase nuclei, and, thus, it showed a greater *H*_coefficient_ (Additional file 1: Fig. S3b). The Pearson’s coefficient ranges between − 1 (anti-correlated signals) to + 1 (correlated signals).

**Fig. 2:**
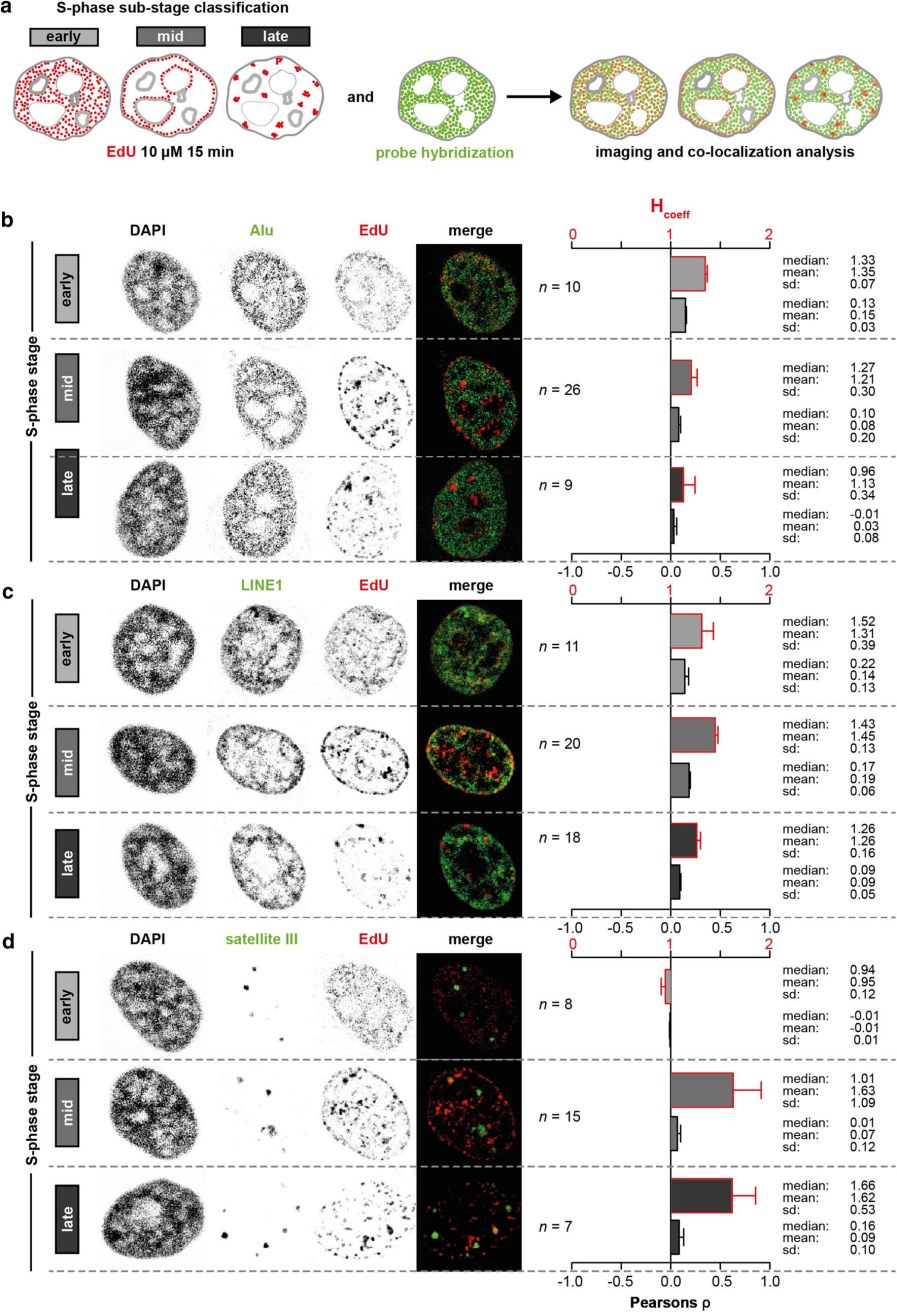
Replication timing of repetitive DNA elements analyzed by FISH and S-phase substages classification. **a** Schematics of the experiment. HeLa cells were pulse-labeled with EdU for 15 min to allow the classification of different substages of the S-phase of the cell cycle (early, mid and late). Cells are then fixed, the probe is hybridized and microscopy is performed. **b** (left) Representative confocal and deconvolved micrographs of HeLa cells depicting the DAPI, Alu elements and EdU as inverted gray channels, at the three different S-phase substages. Merge is shown in pseudo-colors. Scale bar: 5 µm. (right) Colocalization analysis of FISH and EdU signal at the three different S-phase substages via H_coefficent_ and Pearson’s correlation coefficient as indicated. Error bars show the standard error of the mean. Data are from three independent experiments. *n* combined total number of cells analyzed. *sd* standard deviation. **c**, **d** Represent the same as in **b** for L1 and satellite III, respectively

The EdU pulse labeling allowed us to identify three different substages of the S-phase [35]: the early S-phase presented nuclear EdU foci distributed throughout the nuclear interior; the mid S-phase mainly showed foci at the peri-nuclear and peri-nucleolar regions; the late S-phase exhibited distinct nuclear spots corresponding to highly compacted chromatin (i.e., heterochromatin) in HeLa cells (Fig. 2a). We validated the S-phase classification using fluorescence staining of incorporated EdU by measuring the total genomic DNA content of the staged cells and comparing with the DNA content of the EdU negative cells in the population. The latter were further subdivided into G1 and G2 based on their nuclear volume and DAPI content. We observed a steadily increase in the DNA content of the S-phase staged cells from early via mid-to-late S-phase cells, with all three populations exhibiting a larger DNA content than G1 cells and a smaller compared to G2 cells (Additional file 1: Fig. S3c). After probe hybridization, cells were imaged, deconvolved and staged according to their S-phase pattern. To measure the colocalization of the EdU and FISH signals, first a nuclear mask was generated based on the DAPI (DNA) channel. Then, to remove background signal, a local mean filter was applied to the channels to be compared. Finally, the Pearson’s and H_coefficient_ were calculated for each z-plane (Additional file 1: Fig. S4a).

Similar to the genomic data, Alu DNA duplication prior to cell division was strongly associated to the early substage of the S-phase and anti-correlated to the late S-phase (Fig. 2b). L1 DNA replication was associated with all the substages of the S-phase (Fig. 2c). Satellite III DNA duplication showed weak anti-correlation with the early S-phase and strong positive correlation with mid and late S-phase, instead (Fig. 2d). We obtained similar results when we probed the major satellite elements in C2C12 mouse cells, whereby the highest colocalization was detected during late S-phase (Additional file 1: Fig. S5; [36]). Both, H and Pearson’s, coefficients showed similar outcomes. Overall, we observed an euchromatin-to-heterochromatin temporal trend, which can be recapitulated by Alu/L1 and satellite III elements. The outcome of the immuno-FISH analysis strongly supports the genomic data on Alu replication kinetics and, to a lesser extent, also the L1 element genomic data, which we demonstrated to be associated with early and late replicating loci in the genome and be rather dictated by the chromatin modifications they are embedded in. In addition, it extends the investigation to satellites, thus highlighting the importance of performing such combined analysis integrating the benefits of both sequencing and hybridization methodologies to achieve a complete genomic coverage.

### Repair of repetitive DNA elements follows an euchromatin-to-heterochromatin spatio-temporal trend

DNA replication is one of the major causes of endogenous DNA double-strand breaks (DSBs) at collapsed replication forks. Repair and resolution of these lesions is paramount for an error-free cell division. Because DNA replication is temporally and spatially organized [32, 37], we next investigated whether the DNA repair of the above-mentioned repetitive DNA elements followed the same trend we observed for the replication.

**Fig. 3:**
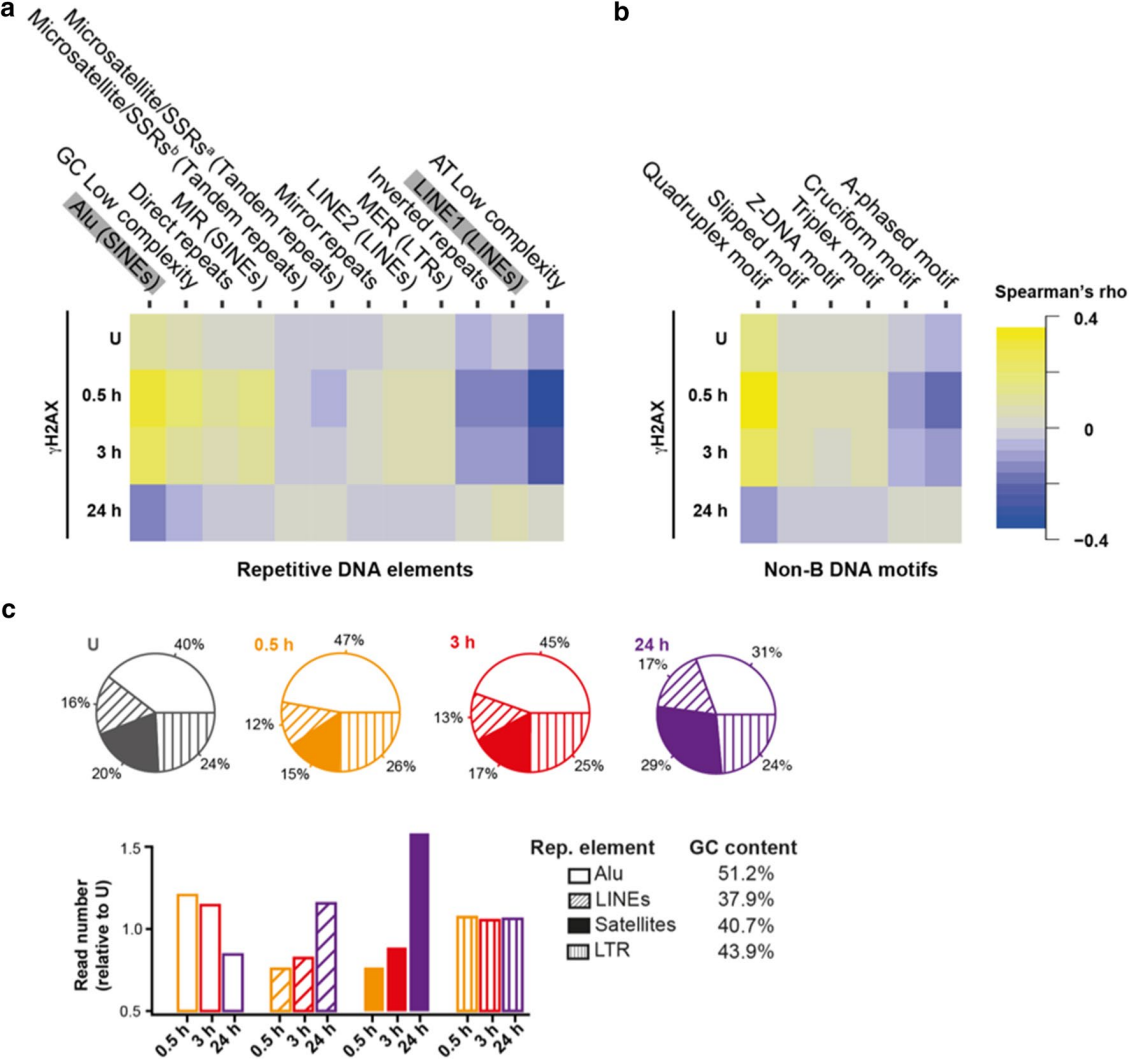
Genome-wide DNA repair kinetics of non-B and repetitive DNA elements. **a** Spearman’s rho correlation matrix between repetitive **a** and non-B **b** DNA elements and γH2AX levels before and after (0.5, 3 and 24 h) IR in HeLa cells. Calculation of the correlation coefficient is as in Fig. 1. **c** (top) Pie-charts showing the distribution of read counts for Alu, LINEs, satellites and LTR repetitive DNA elements, before and after (0.5, 3 and 24 h) IR. (bottom) Bar-plots showing the relative enrichment for the repeat element indicated after (0.5, 3 and 24 h) IR. The number of reads for a given repetitive element and at a given time point was normalized over the corresponding number of reads before IR (for details see Materials and methods section). The respective GC content of the repeat is indicated (whole human genome GC content is 43%)

To evaluate the cellular DNA damage response (DDR) after exposure to ionizing radiation (IR), we assessed the genomic distribution of histone H2AX phosphorylation (γH2AX) by ChIP-Seq at early (0.5 h), mid (3 h) and late (24 h) time points in HeLa cells. Similarly to the analysis of the previously described histone modifications, we computed γH2AX abundance in 10 kbp genomic intervals. Then, we calculated the Spearman’s correlation coefficient between γH2AX abundance and the number of the above-mentioned repetitive DNA elements per genomic interval. The outcome of this analysis indicated that γH2AX was enriched within genomic sequences in which Alu, GC low complexity repeats and direct repeats were abundant (Fig. 3a) at early time points after irradiation. Conversely, AT low complexity repeats and L1 elements presented a lower γH2AX signal (Fig. 3a). This trend was inverted at 24 h post-IR for most of the probed repetitive elements. Also, the abundance of the substrate histone (H2AX) was not relevant for the outcome of the analysis, as only minor differences were observed by comparing input DNA-normalized and H2AX-normalized data (Additional file 1: Fig. S6). Sample genomic loci with tracks showing the repetitive elements and the γH2AX density over the time of the DNA damage response are shown in Additional file 1: Fig. S7.

A large number of repetitive DNA elements can exist in at least two conformations: B and non-B forms. These structures—especially the latter—are involved in genome susceptibility to DNA damage [28]. To investigate which non-B DNA structure was more likely to be formed by a given repetitive DNA element, we first retrieved the genomic distribution of six non-B DNA motifs (cruciform, slipped, triplex, G-quadruplex, Z-DNA and A-phased) from “non-B DB” (https://nonb-abcc.ncifcrf.gov/)—a database for integrated annotation and analysis of non-B DNA forming motifs—and, next, we computed the number of such structures in 10 kbp genomic intervals. Then, we calculated the Spearman’s correlation coefficient between the non-B forms and the previously investigated repetitive DNA elements (Additional file 1: Fig. S8). Microsatellites were strongly associated with Z-DNA and slipped motifs, while inverted repeats showed no correlation with triplex and Z-DNA motifs, but were highly correlated with cruciform motifs (Additional file 1: Fig. S8).

Interestingly, A-phased motifs—formed at A-rich tracts and involved in helix bending and transcription regulation [38, 39]—showed a complex relation with interspersed repetitive DNA elements: Alu and L1 elements were positively correlated with A-phased motifs, yet their respective cognates, MIR and L2, showed a negative correlation (Additional file 1: Fig S8).

Next, we correlated the abundance of γH2AX with the retrieved non-B DNA motifs, over time. In the absence of IR, the endogenous γH2AX signal was slightly correlated with G-quadruplex motifs (Fig. 3b). The latter presented the highest γH2AX levels directly after irradiation (Fig. 3b). Cruciform motifs, which are mainly formed by inverted and direct repeats and are associated with H3K9me3, presented low γH2AX signal, compared to G-quadruplex. Slipped motifs, which are also found in inverted repeats-rich genomic regions, presented no correlation with γH2AX, instead. This divergent behavior highlights the complexity of the genomic compartmentalization and collocates slipped motifs in regions of chromatin responding to DNA DSB signaling at earlier stages of DDR.

We observed yet another divergent behavior pertaining to the Alu and L1 elements. Both elements are positively associated to A-phased motifs, yet the latter are negatively correlated with γH2AX (Fig. 3b). This observation may underline a specific regulation of DDR in A-phased-rich regions.

To measure the total γH2AX signal mapped to Alu, LINEs, satellites and LTRs repetitive elements in an unbiased fashion, we made use of the raw sequence data, as these elements are not filtered for unique mappability. First, we mapped the quality-filtered raw reads to the corresponding repetitive elements as annotated in RepeatMasker. Then, the number of reads in each class was normalized to the total number of repetitive elements reads, containing only signatures of a single repetitive element type, so that the resulting fraction represents the genome-wide γH2AX coverage in a given repetitive element, which we deemed “metarepetitive element.” The analysis of the metarepetitive elements revealed that the Alu signature increased by about 7% at 0.5 h post-IR (47%), with a concomitant decrease in the LINEs (− 4%) and satellites (− 5%) signatures, compared to the unirradiated control (Fig. 3c). Conversely, 24 h post-IR the Alu signature decreased to 31%, whereas the satellites signature increased from 15 to 29% (compared to 0.5 h post-IR). The read counts containing LTR signature mainly remained unvaried (Fig. 3c).

### FISH-based repair timing assessment of interspersed and tandem repeats

We, then, investigated the DDR in repetitive elements by means of immuno-FISH, as we did for replication. Because H2AX distribution can affect, per se, the spreading of γH2AX, we first performed a correlation analysis between the H2AX histone distribution and the Alu, L1 or satellite III DNA repetitive elements using the *H*_coefficient_ and the Pearson’s coefficient. HeLa cells were fixed and immunostained for H2AX, and then incubated with Alu/L1/satellite III probes for the hybridization. The analysis was then performed as previously described (Additional file 1: Fig. S4b). While Alu and L1 showed a clear positive correlation with H2AX histone distribution, satellite III signal showed no correlation with H2AX distribution (Additional file 1: Fig. S9). This is in line with the outcome of the genomic data.

**Fig. 4:**
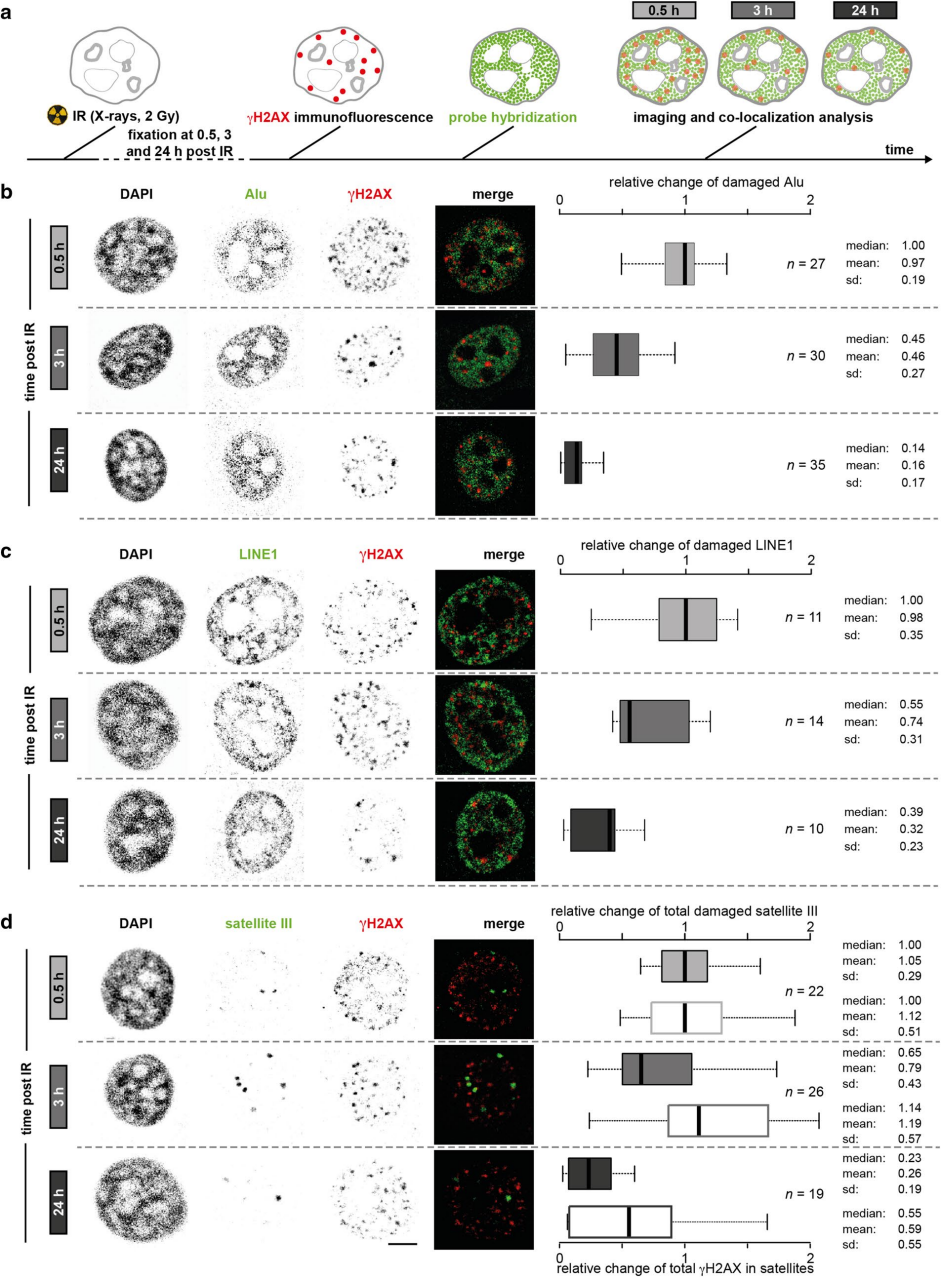
DNA repair kinetics of repetitive DNA elements assessed by FISH. **a** Schematics of the experiment. HeLa cells were sham-irradiated or irradiated with 2 Gy X-rays and incubated for 0.5, 3 and 24 h. γH2AX immunofluorescence and probe hybridization were performed before confocal micrographs acquisition and deconvolution. **b** (left) Representative confocal micrographs of HeLa cells depicting the DAPI, Alu elements and EdU as inverted gray channels, at the three time points post-IR. Merge is shown in pseudo-colors. Scale bar: 5 µm. (right) Relative change of Alu fraction in γH2AX foci. Data are normalized to the median of the 0.5 h time point. Boxes represent median, 2nd and 3rd quartile. Whiskers indicate three times the interquartile distance. Data are from three independent experiments. *n* combined total number of cells analyzed. *sd* standard deviation. **c**, **d** Represent the same as in **b** for LINE1 and satellite III, respectively. In **d**, the empty boxes represent the relative change of γH2AX intensity in segmented satellite III regions

Next, we investigated γH2AX response and its association with Alu, L1 or satellite III DNA repetitive elements. The cells were irradiated with 2 Gy X-rays and incubated for 0.5, 3 or 24 h post-IR, to recapitulate the early, mid and late stages of DDR (Fig. 4a). Cells were fixed at the indicated times and immunostained for γH2AX followed by hybridization with Alu/L1/satellite III probes. The analysis was performed as previously described, with the exception that the first image mask was built from the γH2AX signal (Additional file 1: Fig. S2b). This allowed us to directly compare the repetitive element and the γH2AX fluorescent signals. The focal γH2AX pattern was segmented, and the fraction of each repetitive element within the segmented γH2AX space was calculated for all time points. This was done by taking the sum of repetitive element intensity values within the segmented γH2AX focal structures divided by the total nuclear intensity of the repetitive element. Data were further normalized to the median of the 0.5 h time point to represent the fold change in the damaged regions and in view of the strong differences in γH2AX levels at the different time points. In the absence of IR, γH2AX signal was low and not sufficient to efficiently run the analysis, and, therefore, it was omitted. Images of unirradiated cells are shown in Additional file 1: Fig. S10.

The highest fraction of damaged DNA is expected at 0.5 h post-IR, with a decrease over the time, as the damage is repaired and γH2AX signature is removed. This behavior was observed for all the repetitive elements investigated. The total fraction of Alu or L1 in γH2AX foci was highest upon exposure to IR (0.5 h) (Fig. 4b, c) and decreased to about 50% at 3 h post-IR. 24 h post-IR, the repetitive element fraction in γH2AX foci was about 14–39% of the original (0.5 h post-IR) (Fig. 4b, c). For satellite III DNA, we observed a delayed kinetics, whereby the fraction at 3 and 24 h post-IR was about 65% and 23%, respectively (Fig. 4d, filled boxes). Because satellite DNA elements present a focal structure, we also segmented the satellite regions and determined the fraction of total γH2AX signal within the segmented regions (Fig. 4d, empty boxes). As Alu and L1 spread over the whole genome/nucleus and thus do not allow efficient segmentation, this reciprocal analysis was not performed. In satellites, the fraction of γH2AX remained unvaried up to 3 h post-IR and decreased to 55% at 24 h. Visual inspection of the images revealed that in many cells, the satellite III regions contain only a few γH2AX foci (with partial overlap due to DNA decondensation) or, vice versa, the majority of γH2AX foci contained only a few satellite III regions with partial overlap. We obtained similar results when we probed the major satellite in C2C12 mouse cells (Additional file 1: Fig. S11a, b).

## Discussion

Taken together, repetitive elements appear to be well integrated into chromatin and are preserved by DNA replication and repair processes with the same fidelity as the rest of the genome. All three repetitive elements examined by immuno-FISH follow the general trend of early replication and repair of euchromatin and later replication and repair of heterochromatin. Such observation is consistent with the trend that euchromatin is repaired faster than heterochromatin [40].

Previous studies employing genome-wide approaches showed a correlation between early replicating regions and enrichment for the interspersed Alu elements, while mid and late replicating regions were enriched in L1 [41]. Our combined genomics and immuno-FISH analyses further refines this conclusion in that L1 elements are found to be enriched from early throughout mid S-phase and, to a lesser degree, late S-phase. Recent findings indicate that L1 elements can modulate replication timing of mammalian chromosomes [42]. In fact, we also find that the presence of L1 elements—and possibly their transcriptional context—perturbs the replication program. Furthermore, we extended the analysis to tandem satellite repeats and showed that they are replicated in mid-to-late S-phase. The megabase-long tandem satellite repeats have been shown to contain replication initiation sites allowing their replication, which would be challenging from single forks emanating from the flanking non-repeated DNA regions [43]. As late replicating/repairing (peri-)centromeric genomic regions are rich in tandem repeats (in the order of hundreds of kilobases), these are not well represented in genome-wide studies. This may lead to a general lack of information on this last stage of the replication/repair processes and, thus, mislabeling as late replicating/repairing regions that are instead mid replicating/repairing. Therefore, immuno-FISH data and genome-wide studies complement each other.

Inverted repeats may form stem-loop structures that are often acknowledged to mediate genome instability through excision of the repeat-associated regions [44]. The same is the case for tandem repeats from satellites, where this chromatin mark and condensed structure has been proposed to play a role in avoiding spurious recombination events as discussed below.

We found that tandem repeats diverged from the global genome repair kinetics and were not repaired until late time points and were replicated in late S-phase. The minor overlap of γH2AX with pericentromeric satellite DNA raised the question if the non-canonical histone H2AX is at all located at these regions. Our correlation analysis indicated that the H2AX signal was not correlated with satellite III, leaving the question open. Nonetheless, previous studies showed how γH2AX signal was “bent” around the heterochromatic regions in human and mouse cells upon irradiation with accelerated charged particles [45], thus relocating the chromatin outside of the original lesion site, toward the interface between heterochromatin and euchromatin. This may explain the low colocalization of γH2AX and satellite DNA signals observed under our experimental conditions. Due to their abundance, satellites may be erroneously utilized as repair templates during homologous recombination. The relocation of the damaged DNA outside of condensed heterochromatin has been proposed to avoid the utilization of the wrong chromosome as a template [46].

Altogether, the present study reemphasizes the need for complementary approaches (such as ChIP-Seq and immuno-FISH) to achieve an integrated and comprehensive investigation of any genomic processes.

## Conclusions

**Fig. 5:**
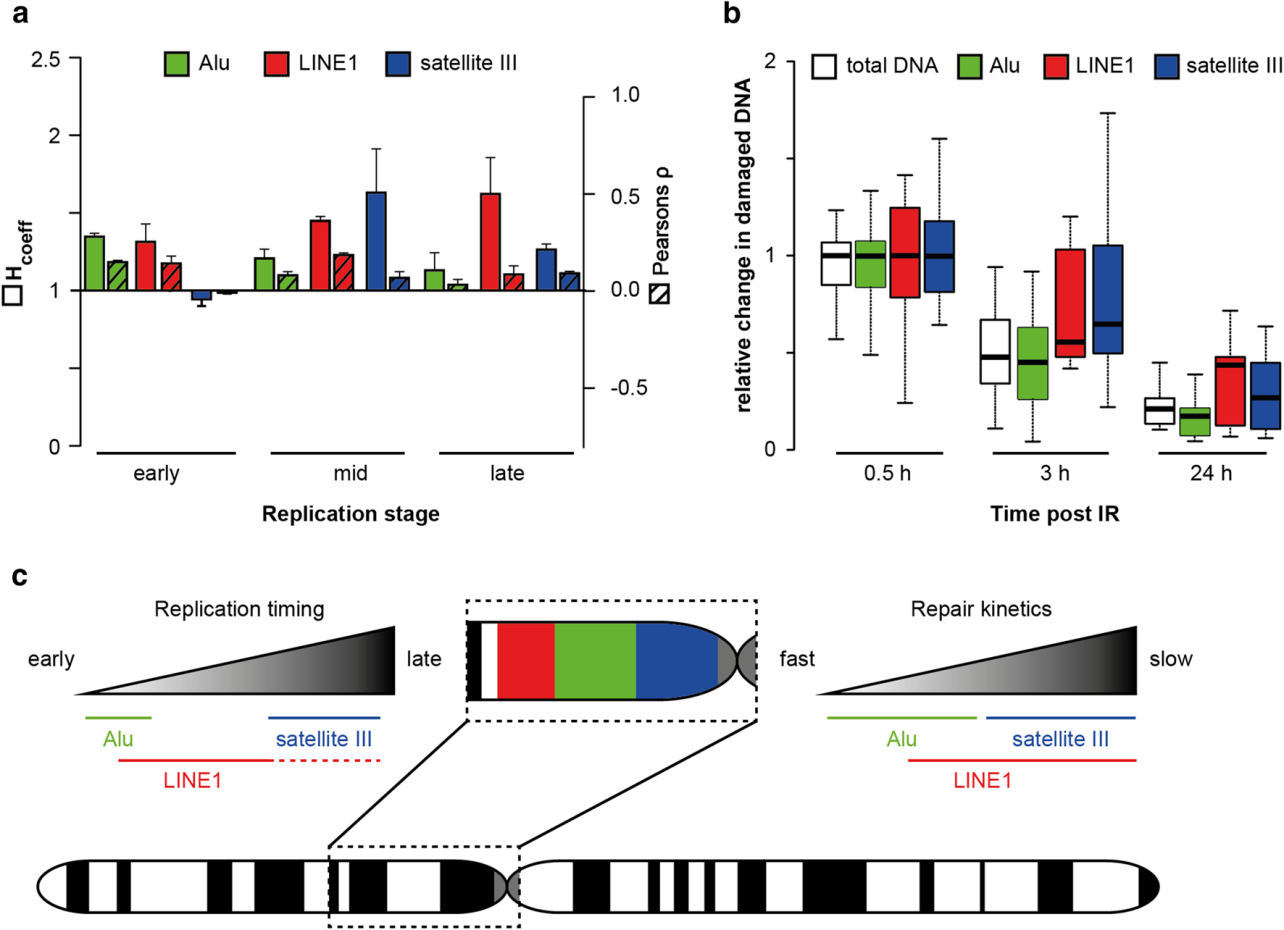
Distribution, replication and repair kinetics of human repetitive elements. **a** Side-by-side comparison of colocalization analysis of repetitive DNA element and DNA replication signals at the three different S-phase substages. **b** Similarly, side-by-side comparison of global genome DNA repair kinetics and each of the different repetitive DNA elements indicated. **c** Graphical summary of the replication and repair kinetics of Alu, L1 and satellite II repetitive elements in the context of their respective chromosomal distribution

The aim of this work was to gain insight into how chromatin and its structural organization influences the genome maintenance processes of DNA replication and repair of repetitive elements. We employed an immuno-FISH approach to simultaneously label replication/repair and three different repetitive DNA elements. We were able to show that (1) the euchromatic Alu element is replicated during early S-phase; (2) L1, which is associated with AT-rich genomic regions, is replicated throughout S-phase, according to the repeat’s particular histone marks; (3) satellite III, which constitutes pericentromeric heterochromatin, is replicated exclusively at the mid-to-late S-phase. These data are summarized in Fig. 5a, c. As for the DNA repair process, we observe that Alu elements are repaired similarly to the total DNA, as observed by the concomitant decrease in the γH2AX signal in Alu chromatin. Furthermore, this mirrors the global genome repair kinetics (Fig. 5b white boxes). Differently, satellite III and L1 elements showed slower repair kinetics, as their γH2AX mark was retained longer (Fig. 5b, c). While the γH2AX response in L1, Alu and satellite elements follows the corresponding GC equivalent of the total genome, this was not the case for LTR, indicating that their γH2AX response may be further affected by other factors.

Based on the repeat’s specific histone marks, we conclude that the histone modifications in the specific repeat element predominantly determine its replication and repair timing. Thus, Alu elements, which are characterized by euchromatic chromatin features, are repaired and replicated the earliest, followed by LINE-1 elements, including more variegated eu/heterochromatic features and, lastly, satellite tandem repeats, which are homogeneously characterized by heterochromatic features and extend over megabase-long genomic regions.

## Methods

### Cell culture and exposure to ionizing radiation

C2C12 mouse myoblasts (ATCC CRL-1772) and HeLa cells (ATCC CCL-2) were grown at 37 °C and 5% CO_2_, in Dulbecco’s modified Eagle’s medium supplemented with 50 µg/mL gentamycin, 20 mM l-glutamine and 10 or 20% fetal calf serum for HeLa and C2C12, respectively. For microscopy-based experiments, cells were grown on glass coverslips. Irradiation was performed with an ISOVOLT Titan E X-ray machine (GE). Cells were exposed to doses of 2–10 Gy (90 kV, 33.7 mA).

### Chromatin immunoprecipitation

HeLa cells were fixed with 1% formaldehyde for 10 min at room temperature, and the crosslink was quenched with 125 mM glycine (5 min at room temperature). Nuclei were isolated after mild lysis in hypotonic buffer (10 mM HEPES pH 8, 1.5 mM MgCl_2_, 60 mM KCl) and 20 strokes in a tight dounce homogenizer. Chromatin was sheared in sonication buffer (0.5% SDS 10 mM EDTA, 50 mM Tris–HCl pH 8.1). Fragmentation of chromatin was carried out by ultrasound treatment (Bioruptor UCD200) so that fragments of 200–300 bp length were obtained. Chromatin from 1–2 × 10^6^ cells was immunoprecipitated with 3 µg mouse anti-γH2AX (Clone JBW301, Upstate) or mouse anti-H2AX (Bethyl Laboratories, A300-83A) antibody. Chromatin was then incubated overnight at 4 °C with protein G-coated magnetic beads (ChIP-IT Express, Active Motif). The chromatin collected (ChIP sample) was then reverse-crosslinked in the presence of 200 mM NaCl at 65 °C for at least five hours, followed by RNase A (50 µg ml^−1^) treatment for 30 min at 37 °C and proteinase K (100 µg ml^−1^) treatment for 3 h at 50 °C. DNA elution was carried out in 1% SDS, 100 mM NaHCO_3_, in a rotary shaker at room temperature for 15 min. Pure DNA was isolated using the Qiagen PCR purification kit, and 15–30 ng of size-selected DNA fragments (Qubit fluorometric quantification) were used to produce ChIP-Seq libraries (Illumina ChIP-Seq DNA sample Prep Kit). Input sample was essentially prepared following the same protocol, but the immunoprecipitation step was skipped.

### Next-generation sequencing (NGS) and data analysis

γH2AX ChIP-Seq libraries were generated and processed as described in [33]. The corresponding datasets are from [33] and can be found at the Gene Expression Omnibus database (GEO accession number: GSE60526). Briefly, reads were mapped to the human genome (University of California, Santa Cruz (UCSC) hg19 assembly, based on the National Center for Biotechnology Information (NCBI) build 37.1) by means of SOAP2 software [47], allowing up to two mismatches for each 36 bp read. Data for genomic features were retrieved from publicly available databases (UCSC/non-B DB) (Table 1). Accession numbers for histone modification ChIP-Seq data are given in Table 2.

**Table 2:** ENCODE datasets used for histone modifications and replication timing

Cell line	Dataset	Data source [48, 49]
HeLa-S3	Repli-seq data	GSM923449
HeLa-S3	H3K4me1	GSM798322
HeLa-S3	H3K4me2	GSM733734
HeLa-S3	H3K4me3	GSM73368
HeLa-S3	H3K36me3	GSM733711
HeLa-S3	H3K79me2	GSM733669
HeLa-S3	H3K9ac	GSM733756
HeLa-S3	H3K27ac	GSM733684
HeLa-S3	H3K27me3	GSM733696
HeLa-S3	H3K9me3	GSM1003480
GM12878	Repli-seq data	GSM923451
GM12878	H3K4me1	GSM733772
GM12878	H3K4me2	GSM733769
GM12878	H3K4me3	GSM733708
GM12878	H3K36me3	GSM733679
GM12878	H3K79me2	GSM733736
GM12878	H3K9ac	GSM733677
GM12878	H3K27ac	GSM733771
GM12878	H3K27me3	GSM733758
GM12878	H3K9me3	GSM733664
HepG2	Repli-seq data	GSM923446
HepG2	H3K4me1	GSM798321
HepG2	H3K4me2	GSM733693
HepG2	H3K4me3	GSM733737
HepG2	H3K36me3	GSM733685
HepG2	H3K79me2	GSM733641
HepG2	H3K9ac	GSM733638
HepG2	H3K27ac	GSM733743
HepG2	H3K27me3	GSM733754
HepG2	H3K9me3	GSM1003519

Data that were originally generated in the hg18 assembly were transposed to hg19 using LiftOver (http://genome.ucsc.edu/cgi-bin/hgLiftOver). Reads per kilobase per million reads (RPKM) [50] were calculated for non-overlapping 10 kb genomic intervals for all sequence tracks. Correlation with γH2AX, Repli-Seq data and genomic features was performed by Spearman’s rho correlation coefficient, with *P* < 2.2 × 10^−16^ in all cases.

Since the majority of reads containing repetitive elements cannot be mapped uniquely, they are usually underrepresented in NGS analysis. To measure the total γH2AX signal mapped to Alu, LINEs, satellites and LTRs repetitive elements in an unbiased fashion, first, we mapped the quality filtered raw reads to human genome, then the reads which uniquely mapped to the corresponding repetitive elements as annotated in RepeatMasker were counted into the corresponding repetitive elements. For the multiple mapped reads, if all mapped genomic positions were annotated as the same class of repetitive element, these reads were still counted into that single repetitive element. If multiple mapped positions were annotated as different type of repetitive elements, these reads were discarded, instead. Finally, the number of reads in each class was normalized to the total number of repetitive elements reads, containing only signatures of a single repetitive element type, so that the resulting fraction represents the genome-wide γH2AX coverage in a given repetitive element, which we deemed “metarepetitive element.” In ChIP-seq data analysis, which covers a minor proportion of the genome, the probability of reading the same sequence twice is higher than in whole-genome sequencing. Hence, de-duplication of PCR artifacts is less critical [51].

### Probe generation for fluorescence in situ hybridization (FISH)

Probes for Alu elements were generated by first amplifying Alu elements from HeLa genomic DNA (gDNA) via PCR using specific Alu primers (AluF: GGATTACAGGYRTGAGCCA; AluR: RCCAYTGCACTCCAGCCTG, [52]), followed by a labeling PCR with the same primers, biotin-labeled dUTP and the previous PCR product (diluted 1:50 in ddH_2_O) as DNA template. The product of the labeling PCR was then purified with QIAquick PCR purification kit (Qiagen). Mouse major satellite (MaSat) probes were generated by PCR using C2C12 gDNA and specific MaSat primers (MaSatF: AAAATGAGAAACATCCACTTG; MaSatR: CCATGATTTTCAGTTTTCTT, [53]).

All PCRs and cycling conditions are listed in Tables 3 and 4.

**Table 3:** PCR conditions for the generation of FISH probes

PCRs	Alu (template)	Alu (labeling)	MaSat
PCR buffer*	1 ×	1 ×	1 ×
dATP/dGTP/dCTP	0.2 mM each	0.2 mM each	0.4 mM each
dTTP	0.2 mM	0.15 mM	–
dUTP-biotin	–	0.05 mM	0.08 mM
primer F/R	1 µM each	1 µM each	0.2 µM each
Taq polymerase	1.5 µL	1.5 µL	1 µL
DNA template	100 ng gDNA	1 µL 1:50 PCR product	100 ng gDNA
Final volume	to 50 µL	to 50 µL	to 50 µL

* 10× PCR buffer: 100 mM Tris–HCl pH 8.3, 500 mM KCl, 15 mM MgCl_2_

**Table 4:** PCR cycling conditions for the generation of FISH probes

PCR cycling condition	Alu	MaSat
Initial denaturation	94 °C for 4 min	98 °C for 10 min
1. Denaturation	94 °C for 1 min	98 °C for 1 min
2. Annealing	57 °C/65 °C for 1 min*	56 °C for 1 min
3. Extension	72 °C for 3:30 min	72 °C for 2 min
No. of cycles steps 1.–3.	35	35
Final extension	72 °C for 10 min	72 °C for 5 min

* Temperature for template PCR/temperature for labeling PCR

Probes for L1 and for chromosome 1 specific satellite III were generated by nick-translation of plasmids containing the corresponding sequences: pLRE3-eGFP ([54] kind gift from John V. Moran) for LRE wild-type L1 element and pUC 1.77 [55] for chromosome 1 satellite III. Both probes were labeled with biotin-labeled dUTP via nick-translation. Conditions for nick-translation were as follows: 50 mM Tris–HCl pH 8, 5 mM MgCl_2_, 0.5 mg/mL BSA, 10 mM beta-mercapto-ethanol, 0.04 mM dUTP-biotin or dUTP-digoxigenin, 0.05 mM dATP/dGTP/dCTP each, 0.32 U DNase I (D5025, Sigma-Aldrich), 10 U Klenow fragment (M0210, NEB), 1 µg plasmid DNA in a total volume of 100 µL. The reaction was incubated for 90 min at 15 °C and stopped with 5 µL 0.5 M EDTA.

All probes were sheared with a Covaris S220 (Covaris Inc.) in microTUBEs (50–65 µL aliquots; 520,045, Covaris Inc.) to a final size of ~ 250 bp. The amount of probe required for FISH was then ethanol-precipitated in the presence of sodium acetate, washed with 70% ethanol, air-dried at room temperature, dissolved in hybridization solution: (1) with formamide (50–70% formamide, 2× SSC, 10% dextran sulfate, pH 7) for Alu and LINE1 FISH on metaphase spreads; (2) without formamide (10 mM Tris–HCl, 3 mM MgCl_2_, 50 mM KCl, 10 µg/mL gelatin, 2× SSC [56, 57] for satellite III and MaSat FISH on metaphase spreads and for interphase FISH. The amounts of probes were as follows: 250, 200 or 50 ng for L1, Alu and satellite III, respectively. 5 µL PCR were used for MaSat. For all probes (except the satellite III probe on interphase cells), 1 µg fish sperm DNA was added. For metaphase or interphase FISH, probes were dissolved in 30 and 15 µL hybridization solution, respectively. Probes were then denatured for five minutes at 80 °C.

### Metaphase spreads preparation and FISH on metaphase chromosomes

HeLa and C2C12 cells were treated with 0.1 µg/mL colcemid for two hours. Cells were then harvested by trypsinization and first incubated for 20 min with 75 mM KCl at room temperature. They were then fixed in dropwise added ice-cold methanol/acetic acid (3:1) for 30 min on ice. The fixation step was repeated twice. For chromosome spreading, the cell suspension was dropped onto a wet microscopy slide from a height of approximately 25 cm. The slide was then air-dried overnight. For metaphase FISH, the slides were rehydrated in ddH_2_O for 10 min, digested with 0.005% pepsin (165 U/mL, P6887, Sigma-Aldrich) in 0.01 M HCl for 10 min at 37 °C, then dehydrated in 70 and 100% ethanol for 5 min each. Finally, the slides were air-dried overnight.

Equilibration of metaphase spreads was performed with the respective hybridization solution (see above) at room temperature for 30 min. The solution was removed, and the probes were combined with the metaphase spreads in a humid chamber. Denaturation was performed at 70–80 °C in a water bath for 5 min and the hybridization followed at 37–42 °C overnight. Post-hybridization washing steps were done with 2× SSC and 0.1× SSC at 42 °C. Slides were blocked with 1% BSA/4× SSC for 30 min and the FISH probes detected with streptavidin Alexa Fluor 488 (S11223, Molecular Probes/Thermo Fisher Scientific, 1:800) or rabbit anti-digoxigenin (Cat#: 700772, Abfinity, 1:500) and anti-rabbit IgG Cy5 (711-175-152, Jackson ImmunoResearch, 1:400) in 1% BSA/4 × SSC for 30 min. DNA counterstaining was performed with DAPI (1 µg/mL) for 10 min and the coverslips were mounted in Mowiol 4-88/2.5% DABCO.

### Combination of replication staining (EdU Click reaction) or immunofluorescence staining of γH2AX with FISH

Cells were pulse-labeled with 10 µM EdU for 15 min or irradiated with 2 Gy X-rays. For replication staining, fixation with 3.7% formaldehyde/1 × PBS followed directly after the pulse-labeling and for irradiated cells 0.5, 3 or 24 h post-IR. Cells were permeabilized and pre-denatured with 0.5% Triton X-100 in 1 × PBS for 15 min, 0.1 M HCl for 15 min and 0.5% Triton X-100/1 × PBS for 15 min.

EdU was detected with the EdU Click-594 ROTI kit (7776.1, Carl Roth), according to manufacturers’ instructions. The dye azide was used in a final dilution of 1:2000.

For immuno-staining of γH2AX, irradiated cells were blocked with 1% BSA/1 × PBS for 30 min, incubated with the primary antibody mouse anti-histone H2AX phospho-Ser139 (clone JBW301, 05-636, Upstate/Millipore, 1:200) in 1% BSA/1 × PBS for 1 h and incubated with the secondary antibody donkey anti-mouse IgG Cy5 (715-175-150, Jackson ImmunoResearch, 1:250) in 1% BSA in 1 × PBS for 1 h. Both stainings were post-fixed with 1% formaldehyde/1 × PBS for 10 min before proceeding with FISH.

The cells were equilibrated with hybridization solution without formamide (composition as described above) at room temperature for 30 min. The solution was removed before combining the probes with the cells in a humid chamber; samples were denatured at 80 °C in a water bath for five minutes and hybridized overnight at 42 °C. Post-hybridization washing steps were done with 2 × SSC and 0.1 × SSC at 42 °C. FISH probes were detected with streptavidin Alexa Fluor 488 (S11223, Molecular Probes/Thermo Fisher Scientific, 1:800) in BSA/4 × SSC for 30 min. DNA counterstaining was performed with DAPI (1 µg/mL) for 10 min and the coverslips mounted in Mowiol 4-88/2.5% DABCO.

### Microscopy

Confocal imaging was performed using a Perkin Elmer VoX-1000 Spinning Disk microscope equipped with a 60 ×/1.4 NA/oil CFI Apochromat TIRF objective, four laser lines (405, 488, 561 and 635 nm) and a Hamamatsu EMCCD camera (C9100-50). The following filter sets were used: excitation: quad-bandpass 405/488/568/640 nm with the matching emission filters for DAPI (445/30 nm), Alexa Fluor 488 (500–548 nm), TRITC (526–623 nm) and Cy5 (664–750 nm). For higher special resolution, images were acquired using a Leica SP5 II laser scanning microscope using a 100 × 1.44 NA HCX PL APO Objective with a pixel size of 86.6 nm and a *z*-spacing of 125 nm for subsequent deconvolution. For imaging the 405, 488, 561 and 633 nm laser line and spectral detection windows of 425–465 nm (DAPI), 495–558 nm (Alexa 488), 600–660 nm (Alexa 594) and 640–705 nm (Cy5) were used. Images were then deconvolved with wavelength specific point spread functions using ImageJ and the Iterative Deconvolution 3D plugin [58]. In addition, a Zeiss Axiovert 200 with a 100 ×/1.4 NA/oil Plan-Apochromat objective was used to image metaphase spreads. Images were recorded using a Zeiss Axiocam mRM, and the following filters were used: DAPI; ex: 350/50 nm; bs: 400 nm; em: 460/50 nm and Alexa Fluor 488: ex: 482/18 nm; bs: 495 nm; 520/28 nm.

### Image analysis for repair kinetics of repetitive elements

Image analysis was performed using the image analysis software Perkin Elmer Volocity 6.3. The following steps in the measurements tab were used to segment γH2AX foci and satellite regions (Additional file 1: Fig. S2): “Find object” (“nucleus”) using the DAPI channel (method “automatic,” minimum object size: 400 µm^3^), fill holes in object, dilate with two numbers of iterations, fill holes in object. “Find object” (“repair foci”) using the Cy5 channel, method “SD” (lower limit: set to “optimal value for all cells within one condition,” minimum object size: 0.3 µm^3^), remove noise from objects with fine filter, separate touching objects (object size guide: 1 µm, filter population: volume > 0.3 µm^3^), exclude “satellite” not touching “nucleus.”

### Colocalization analysis for replication timing of repetitive elements

To analyze at which S-phase stage any given repetitive elements are replicated, the cells were categorized into early, mid, or late replication patterns based on EdU signal [35]. The degree of colocalization was scored by the Pearson’s correlation coefficient and the *H*_coefficient_ [34]. First, a nuclear mask was derived from the DAPI channel using ImageJ (Gaussian blur with sigma = 1). Then, a local mean filter was applied (using the platform for image analysis Priithon) to the channels that are to be compared. This removes the background. Next, the *H*_coefficient_ and the Pearson’s coefficient *r* were calculated for each plane. For the plots, a mid-nuclear section was selected from each image as having the best signal quality. The method is schematically summarized in Additional file 1: Fig. S2.

## **Additional file**

**Additional file 1: Figure S1.** Genomic DNA repetitive elements, DNA replication and histone modifications distributions. **Figure S2**. Genome-wide correlation of DNA replication and histone modifications distributions in multiple cell lines. **Figure S3**. FISH probes and correlation analysis validation. **Figure S4**. Image analysis flowchart. **Figure S5**. Replication timing of murine major satellite DNA elements by FISH and S-phase sub-stages classification. **Figure S6**. Genome-wide correlation of DNA repetitive elements and histone γH2AX in HeLa cells. **Figure S7**. Genomic repetitive and non-B DNA elements, and γH2AX histone distributions.** Figure S8**. Relation of repetitive DNA elements to non-B DNA elements. **Figure S9**. Correlation of histone H2AX and repetitive DNA elements before and during the DDR by FISH. **Figure S10**. Complete DNA repair kinetics of repetitive DNA elements analyzed by FISH. **Figure S11**. DNA repair kinetics of murine major satellite DNA elements analyzed by FISH.

## Abbreviations

ChIP-Seq: chromatin immunoprecipitation-massively parallel DNA sequencing
DAPI: 4′,6-diamidino-2-phenylindole
DDR: DNA damage response
FISH: fluorescence in situ hybridization
GEO: Gene Expression Omnibus Database
HR: homologous recombination
IR: ionizing radiation
LINE: long interspersed nuclear element
MaSat: mouse major satellite
NGS: next-generation sequencing
PCR: polymerase chain reaction
RPKM: reads per kilobase per million reads
SINE: short interspersed nuclear element
UV: ultraviolet light

## References

[1] Slotkin RK, Martienssen R (2007). Transposable elements and the epigenetic regulation of the genome. Nat Rev Genet.

[2] Lander ES, Linton LM, Birren B, Nusbaum C, Zody MC, Baldwin J, Devon K, Dewar K, Doyle M, FitzHugh W (2001). Initial sequencing and analysis of the human genome. Nature.

[3] Waterston RH, Lindblad-Toh K, Birney E, Rogers J, Abril JF, Agarwal P, Agarwala R, Ainscough R, Alexandersson M, Mouse Genome Sequencing C (2002). Initial sequencing and comparative analysis of the mouse genome. Nature.

[4] Beck CR, Garcia-Perez JL, Badge RM, Moran JV (2011). LINE-1 elements in structural variation and disease. Annu Rev Genom Hum Genet.

[5] Feng Q, Moran JV, Kazazian HH, Boeke JD (1996). Human L1 retrotransposon encodes a conserved endonuclease required for retrotransposition. Cell.

[6] Khazina E, Truffault V, Buttner R, Schmidt S, Coles M, Weichenrieder O (2011). Trimeric structure and flexibility of the L1ORF1 protein in human L1 retrotransposition. Nat Struct Mol Biol.

[7] Martin SL, Branciforte D, Keller D, Bain DL (2003). Trimeric structure for an essential protein in L1 retrotransposition. Proc Natl Acad Sci U S A.

[8] Mathias SL, Scott AF, Kazazian HH, Boeke JD, Gabriel A (1991). Reverse transcriptase encoded by a human transposable element. Science.

[9] Hohjoh H, Singer MF (1996). Cytoplasmic ribonucleoprotein complexes containing human LINE-1 protein and RNA. EMBO J.

[10] Ostertag EM, Kazazian HH (2001). Biology of mammalian L1 retrotransposons. Annu Rev Genet.

[11] Korenberg JR, Rykowski MC (1988). Human genome organization: Alu, lines, and the molecular structure of metaphase chromosome bands. Cell.

[12] Quentin Y (1994). A master sequence related to a free left Alu monomer (FLAM) at the origin of the B1 family in rodent genomes. Nucleic Acids Res.

[13] Deininger P (2011). Alu elements: know the SINEs. Genome Biol.

[14] Dewannieux M, Esnault C, Heidmann T (2003). LINE-mediated retrotransposition of marked Alu sequences. Nat Genet.

[15] Burwinkel B, Kilimann MW (1998). Unequal homologous recombination between LINE-1 elements as a mutational mechanism in human genetic disease. J Mol Biol.

[16] White TB, Morales ME, Deininger PL (2015). Alu elements and DNA double-strand break repair. Mob Genet Elements.

[17] Hancks DC, Kazazian HH (2016). Roles for retrotransposon insertions in human disease. Mob DNA.

[18] Lee E, Iskow R, Yang L, Gokcumen O, Haseley P, Luquette LJ, Lohr JG, Harris CC, Ding L, Wilson RK (2012). Landscape of somatic retrotransposition in human cancers. Science.

[19] Scott EC, Gardner EJ, Masood A, Chuang NT, Vertino PM, Devine SE (2016). A hot L1 retrotransposon evades somatic repression and initiates human colorectal cancer. Genome Res.

[20] Guenatri M, Bailly D, Maison C, Almouzni G (2004). Mouse centric and pericentric satellite repeats form distinct functional heterochromatin. J Cell Biol.

[21] Jarmuz M, Glotzbach CD, Bailey KA, Bandyopadhyay R, Shaffer LG (2007). The Evolution of satellite III DNA subfamilies among primates. Am J Hum Genet.

[22] Waye JS, Willard HF (1986). Structure, organization, and sequence of alpha satellite DNA from human chromosome 17: evidence for evolution by unequal crossing-over and an ancestral pentamer repeat shared with the human X chromosome. Mol Cell Biol.

[23] Wong AK, Rattner JB (1988). Sequence organization and cytological localization of the minor satellite of mouse. Nucleic Acids Res.

[24] Wu JC, Manuelidis L (1980). Sequence definition and organization of a human repeated DNA. J Mol Biol.

[25] Jolly C, Metz A, Govin J, Vigneron M, Turner BM, Khochbin S, Vourc’h C (2004). Stress-induced transcription of satellite III repeats. J Cell Biol.

[26] Saksouk N, Simboeck E, Dejardin J (2015). Constitutive heterochromatin formation and transcription in mammals. Epigenetics Chromatin.

[27] Valgardsdottir R, Chiodi I, Giordano M, Rossi A, Bazzini S, Ghigna C, Riva S, Biamonti G (2008). Transcription of Satellite III non-coding RNAs is a general stress response in human cells. Nucleic Acids Res.

[28] Bacolla A, Wells RD (2004). Non-B DNA conformations, genomic rearrangements, and human disease. J Biol Chem.

[29] Cer RZ, Bruce KH, Mudunuri US, Yi M, Volfovsky N, Luke BT, Bacolla A, Collins JR, Stephens RM (2011). Non-B DB: a database of predicted non-B DNA-forming motifs in mammalian genomes. Nucleic Acids Res.

[30] RepeatMasker Open-4.0 [http://www.repeatmasker.org/].

[31] Hansen RS, Thomas S, Sandstrom R, Canfield TK, Thurman RE, Weaver M, Dorschner MO, Gartler SM, Stamatoyannopoulos JA (2010). Sequencing newly replicated DNA reveals widespread plasticity in human replication timing. Proc Natl Acad Sci U S A.

[32] Chagin VO, Stear JH, Cardoso MC (2010). Organization of DNA replication. Cold Spring Harb Perspect Biol.

[33] Natale F, Rapp A, Yu W, Maiser A, Harz H, Scholl A, Grulich S, Anton T, Horl D, Chen W (2017). Identification of the elementary structural units of the DNA damage response. Nat Commun.

[34] Herce HD, Casas-Delucchi CS, Cardoso MC (2013). New image colocalization coefficient for fluorescence microscopy to quantify (bio-)molecular interactions. J Microsc.

[35] Chagin VO, Casas-Delucchi CS, Reinhart M, Schermelleh L, Markaki Y, Maiser A, Bolius JJ, Bensimon A, Fillies M, Domaing P (2016). 4D Visualization of replication foci in mammalian cells corresponding to individual replicons. Nat Commun.

[36] Casas-Delucchi CS, van Bemmel JG, Haase S, Herce HD, Nowak D, Meilinger D, Stear JH, Leonhardt H, Cardoso MC (2012). Histone hypoacetylation is required to maintain late replication timing of constitutive heterochromatin. Nucleic Acids Res.

[37] Jackson DA, Pombo A (1998). Replicon clusters are stable units of chromosome structure: evidence that nuclear organization contributes to the efficient activation and propagation of S phase in human cells. J Cell Biol.

[38] Jansen A, van der Zande E, Meert W, Fink GR, Verstrepen KJ (2012). Distal chromatin structure influences local nucleosome positions and gene expression. Nucleic Acids Res.

[39] Koo HS, Wu HM, Crothers DM (1986). DNA bending at adenine. thymine tracts. Nature.

[40] Goodarzi AA, Noon AT, Deckbar D, Ziv Y, Shiloh Y, Lobrich M, Jeggo PA (2008). ATM signaling facilitates repair of DNA double-strand breaks associated with heterochromatin. Mol Cell.

[41] Woodfine K, Fiegler H, Beare DM, Collins JE, McCann OT, Young BD, Debernardi S, Mott R, Dunham I, Carter NP (2004). Replication timing of the human genome. Hum Mol Genet.

[42] Platt EJ, Smith L, Thayer MJ (2018). L1 retrotransposon antisense RNA within ASAR lncRNAs controls chromosome-wide replication timing. J Cell Biol.

[43] Erliandri I, Fu H, Nakano M, Kim JH, Miga KH, Liskovykh M, Earnshaw WC, Masumoto H, Kouprina N, Aladjem MI, Larionov V (2014). Replication of alpha-satellite DNA arrays in endogenous human centromeric regions and in human artificial chromosome. Nucleic Acids Res.

[44] Gordenin DA, Lobachev KS, Degtyareva NP, Malkova AL, Perkins E, Resnick MA (1993). Inverted DNA repeats: a source of eukaryotic genomic instability. Mol Cell Biol.

[45] Jakob B, Splinter J, Conrad S, Voss KO, Zink D, Durante M, Lobrich M, Taucher-Scholz G (2011). DNA double-strand breaks in heterochromatin elicit fast repair protein recruitment, histone H2AX phosphorylation and relocation to euchromatin. Nucleic Acids Res.

[46] Tsouroula K, Furst A, Rogier M, Heyer V, Maglott-Roth A, Ferrand A, Reina-San-Martin B, Soutoglou E (2016). Temporal and spatial uncoupling of DNA double strand break repair pathways within mammalian heterochromatin. Mol Cell.

[47] Li R, Yu C, Li Y, Lam TW, Yiu SM, Kristiansen K, Wang J (2009). SOAP2: an improved ultrafast tool for short read alignment. Bioinformatics.

[48] Pope BD, Ryba T, Dileep V, Yue F, Wu W, Denas O, Vera DL, Wang Y, Hansen RS, Canfield TK (2014). Topologically associating domains are stable units of replication-timing regulation. Nature.

[49] Ernst J, Kheradpour P, Mikkelsen TS, Shoresh N, Ward LD, Epstein CB, Zhang X, Wang L, Issner R, Coyne M (2011). Mapping and analysis of chromatin state dynamics in nine human cell types. Nature.

[50] Mortazavi A, Williams BA, McCue K, Schaeffer L, Wold B (2008). Mapping and quantifying mammalian transcriptomes by RNA-Seq. Nat Methods.

[51] Ebbert MT, Wadsworth ME, Staley LA, Hoyt KL, Pickett B, Miller J, Duce J (2016). Alzheimer’s Disease Neuroimaging I, Kauwe JS, Ridge PG: evaluating the necessity of PCR duplicate removal from next-generation sequencing data and a comparison of approaches. BMC Bioinform.

[52] Liu P, Siciliano J, Seong D, Craig J, Zhao Y, de Jong PJ, Siciliano MJ (1993). Dual Alu polymerase chain reaction primers and conditions for isolation of human chromosome painting probes from hybrid cells. Cancer Genet Cytogenet.

[53] Frauer C, Rottach A, Meilinger D, Bultmann S, Fellinger K, Hasenoder S, Wang M, Qin W, Soding J, Spada F, Leonhardt H (2011). Different binding properties and function of CXXC zinc finger domains in Dnmt1 and Tet1. PLoS ONE.

[54] Garcia-Perez JL, Morell M, Scheys JO, Kulpa DA, Morell S, Carter CC, Hammer GD, Collins KL, O’Shea KS, Menendez P, Moran JV (2010). Epigenetic silencing of engineered L1 retrotransposition events in human embryonic carcinoma cells. Nature.

[55] Cooke HJ, Hindley J (1979). Cloning of human satellite III DNA: different components are on different chromosomes. Nucleic Acids Res.

[56] Celeda D, Aldinger K, Haar FM, Hausmann M, Durm M, Ludwig H, Cremer C (1994). Rapid fluorescence in situ hybridization with repetitive DNA probes: quantification by digital image analysis. Cytometry.

[57] Celeda D, Bettag U, Cremer C (1992). A simplified combination of DNA probe preparation and fluorescence in situ hybridization. Z Naturforsch C.

[58] Dougherty RP. Extensions of DAMAS and benefits and limitations of deconvolution in beamforming. In: AIAA 2005-2961; 2005.

