# Supplementary material for: DNA replication and repair kinetics of Alu, LINE-1 and satellite III genomic repetitive elements
Source: Epigenetics Chromatin. 2018 Oct 23;11:61. doi: 10.1186/s13072-018-0226-9 (PMC6198450; doi:10.1186/s13072-018-0226-9)
Supplement: Supplementary file 1 — Additional file 1: Figure S1. Genomic DNA repetitive elements, DNA replication and histone modifications distributions. Figure S2. Genome-wide correlation of DNA replication and histone modifications distributions in multiple cell lines. Figure S3. FISH probes and correlation analysis validation. Figure S4. Image analysis flowchart. Figure S5. Replication timing of murine major satellite DNA elements by FISH and S-phase sub-stages classification. Figure S6. Genome-wide correlation of DNA repetitive elements and histone γH2AX in HeLa cells. Figure S7. Genomic repetitive and non-B DNA elements, and γH2AX histone distributions. Figure S8. Relation of repetitive DNA elements to non-B DNA elements. Figure S9. Correlation of histone H2AX and repetitive DNA elements before and during the DDR by FISH. Figure S10. Complete DNA repair kinetics of repetitive DNA elements analyzed by FISH. Figure S11. DNA repair kinetics of murine major satellite DNA elements analyzed by FISH. [file 13072_2018_226_MOESM1_ESM.docx]

**SUPPLEMENTARY MATERIAL**

**
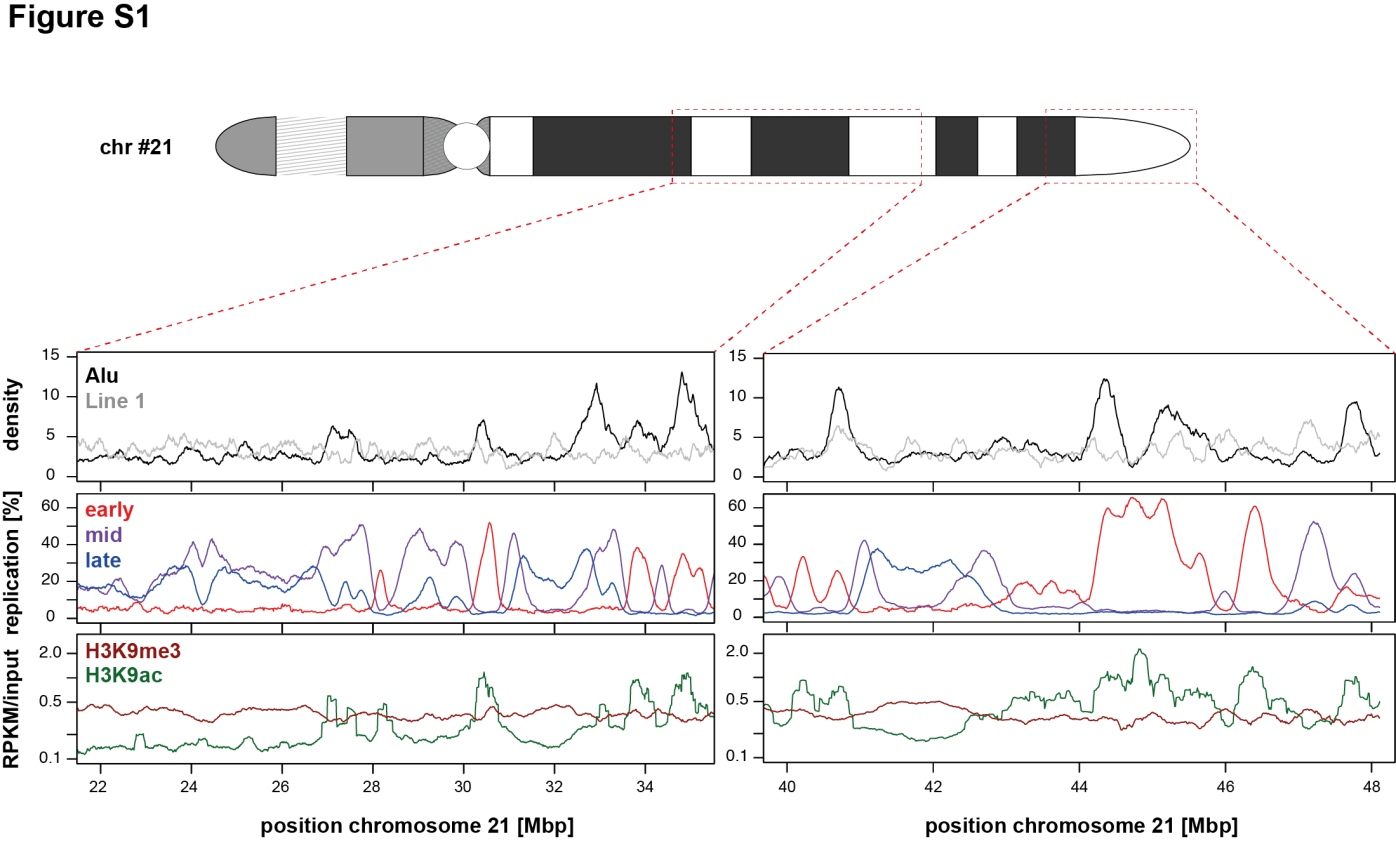
**

**Figure S1. Genomic DNA repetitive elements, DNA replication and histone modifications distributions.** Exemplary genomic (top) Alu and LINE1, (mid) Repli-Seq and (bottom) H3K9me3/ac distributions in 10 kbp intervals from HeLa cells. For Alu and LINE1 elements, the number of elements per interval is shown. All tracks were smoothed using a moving average of 10 intervals.

**
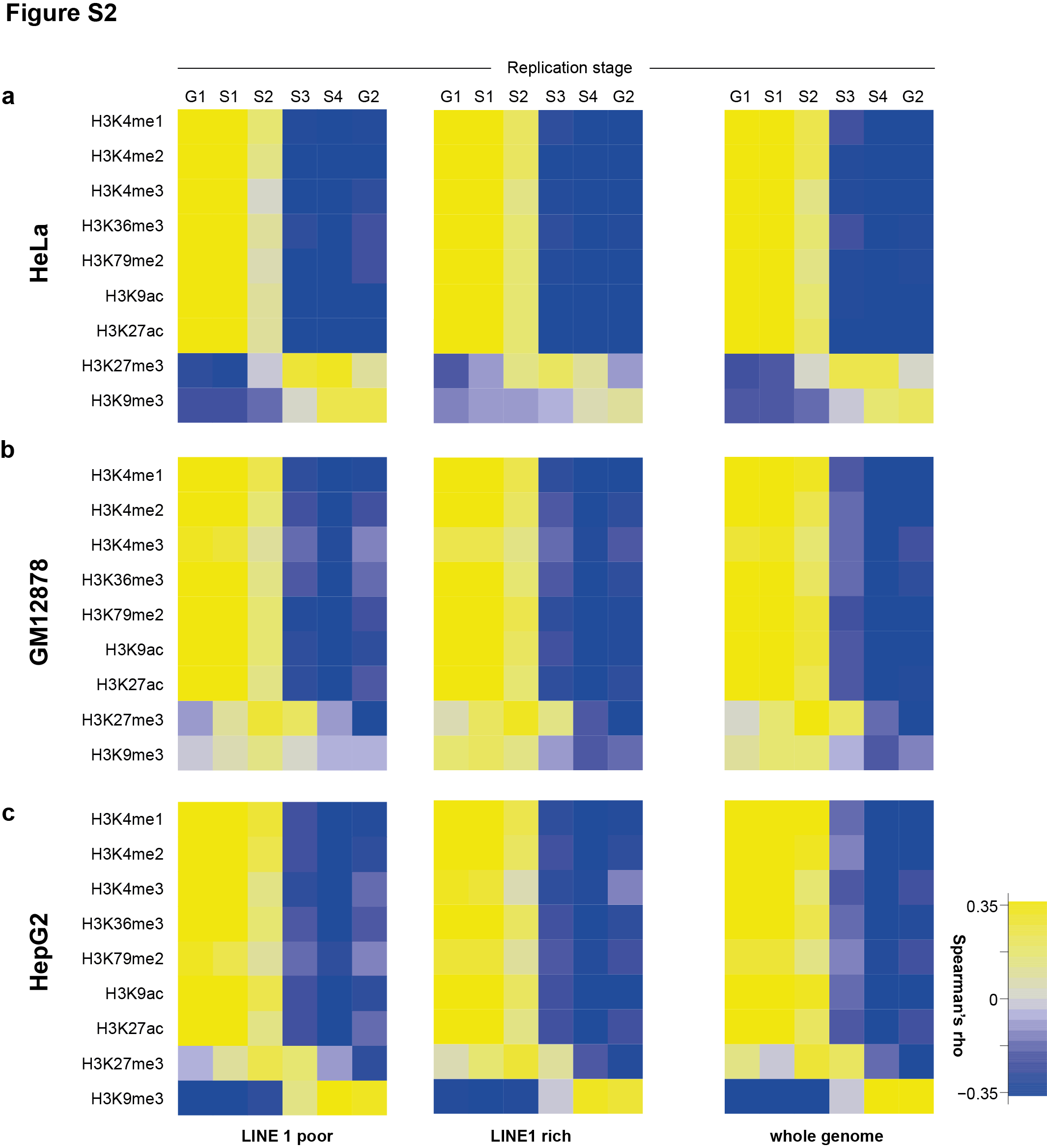
**

**Figure S2. Genome-wide correlation of DNA replication and histone modifications distributions in multiple cell lines.** Spearman’s rho correlation matrix between replication stage (Repli-Seq) and histone modifications (ChIP-Seq) in uterine cervix cancer (HeLa, a), lymphoblastoid (GM12878, b) and hepatocarcinoma (HepG2, c) cell lines. Comparison between LINE1-poor (<1 count per genomic interval, left), LINE1-rich (>10 counts per genomic interval, middle) and whole-genome (right) are shown. For whole-genome correlation, data are from >290,000 genomic intervals with P < 2.2 × 10^-16^. For LINE1-rich/poor correlation matrices, data are from >10,000 genomic intervals with P < 10^-9^.

**
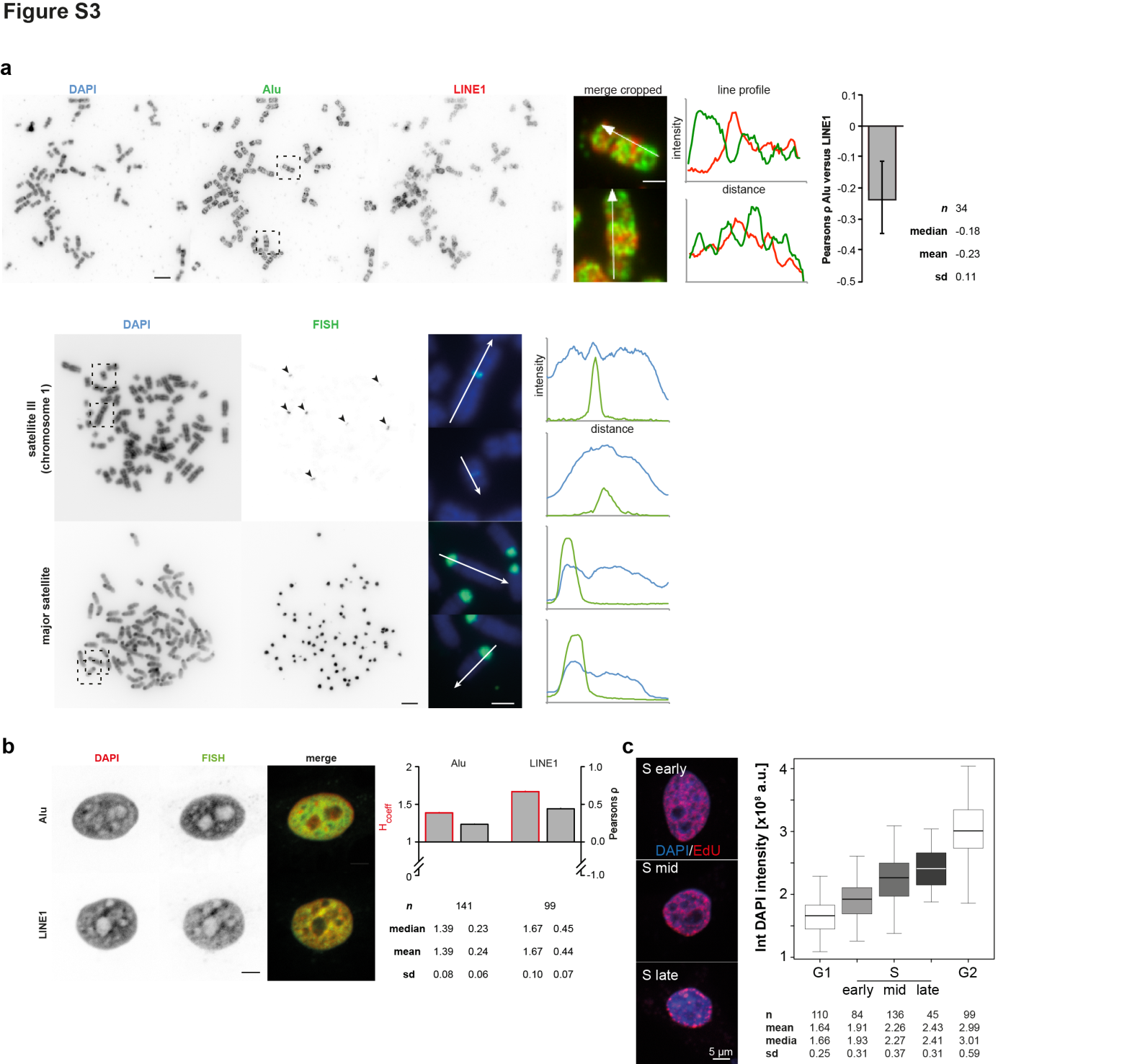
**

**Figure S3: FISH probes and correlation analysis validation.** (a) Double metaphase FISH for Alu and L1 and metaphase FISH for satellite III (chromosome 1) in HeLa cells and major satellite in C2C12 cells. Cell cultures were enriched for mitotic cells by colcemid treatment and after metaphase preparation spread onto glass slides. FISH for Alu and L1 was performed under stringent conditions (with formamide) whereas FISH for satellite III and major satellite was performed under non-stringent conditions (without formamide). Arrowheads point to the hybridized regions of satellite III FISH. Dashed frames are magnified in the merged cropped images. Arrows in the merged images show the position and direction of the line profiles (blue: DAPI; green and red: FISH). Pearson’s correlation factor was calculated for 34 double hybridized chromosomes. Scale bar: 5 µm and 2 µm in full and cropped images, respectively. (b) Colocalization of DAPI with Alu and L1 elements in HeLa cells. (left) Representative images of DAPI and Alu/L1 channels. Merged images are shown in pseudo-colors. Scale bar: 5 µm. (right) Colocalization analysis of FISH and DAPI signal at the three different S-phase sub-stages using H_coefficent_ and Pearson’s correlation coefficient as indicated. Error bars show the standard error of the mean. Data are from three independent experiments. *n*: combined total number of cells analyzed. sd: standard deviation. (c) Verification of S-phase classification using the integrated nuclear DAPI intensity. Left: exemplary images of early, mid and late S-phase cells classified based on immunofluorescence staining of replication patterns where EdU (active replication sites) is depicted in red and DAPI (DNA) in blue. The total nuclear DAPI intensity of cells that were thus classified into early, mid or late S-phase was measured and compared to EdU negative cells with small nuclei (<500 µm^3^) (G1 cells) and large nuclei (>750 µm^3^) (G2 cells).

**
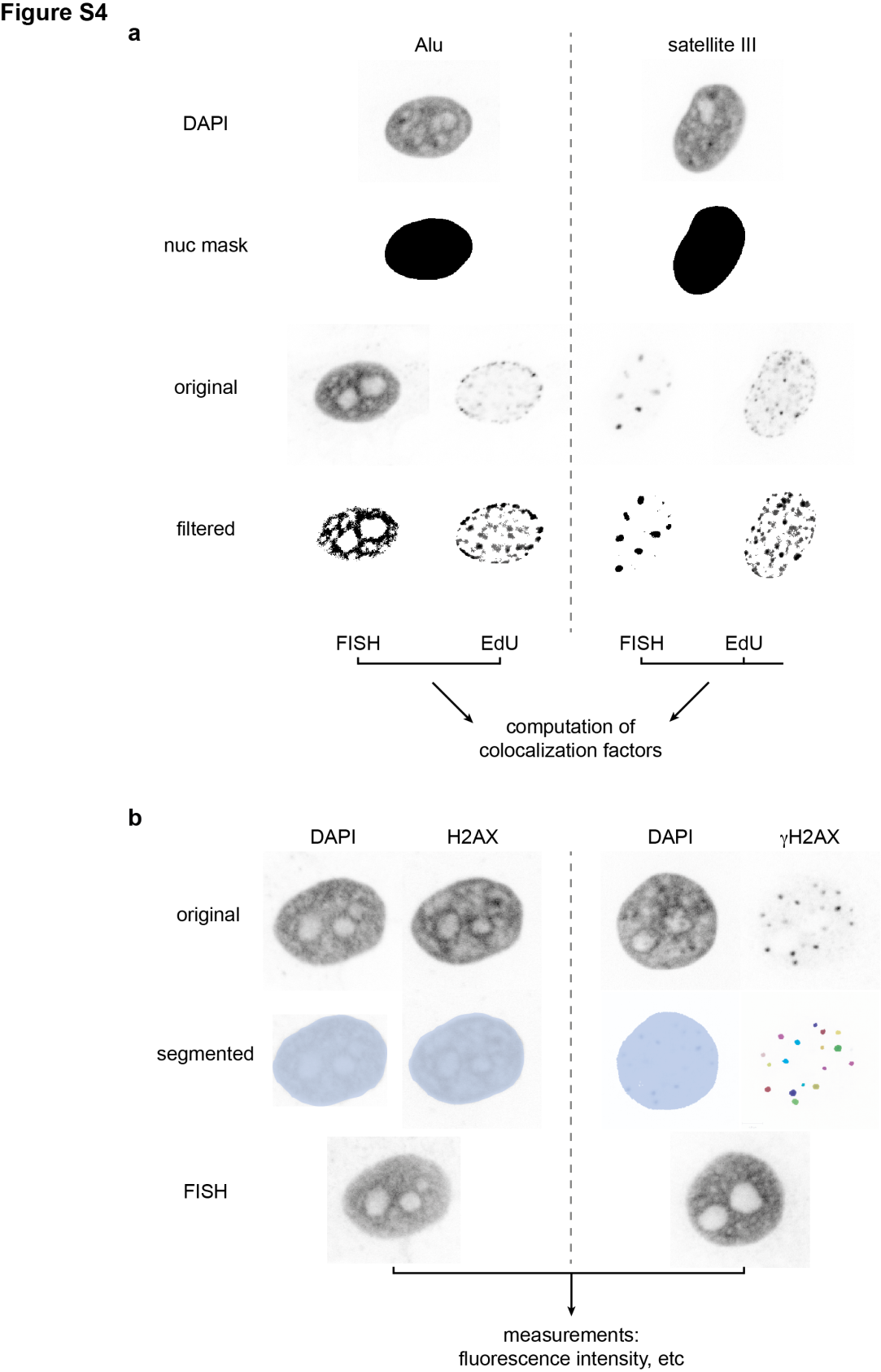
**

**Figure S4: Image analysis flowchart.** (a) Segmentation of DNA repetitive elements and colocalization analysis. First, the DAPI channel is used to generate the nuclear mask (nuc). The other channels are cropped to the “nuc mask” while maintaining the original intensity values. All intensity values outside the “nuc mask” are set to zero. Colocalization factors are then calculated from the filtered and masked images. (b) Segmentation of (γ)H2AX signal. The DAPI channel is used to segment the nucleus. Within the nucleus, H2AX fluorescence was used or γH2AX foci were segmented (different colors refer to different objects). Objects outside the nucleus are excluded. Sum intensity values from all channels are then measured within the segmented focus/nucleus.


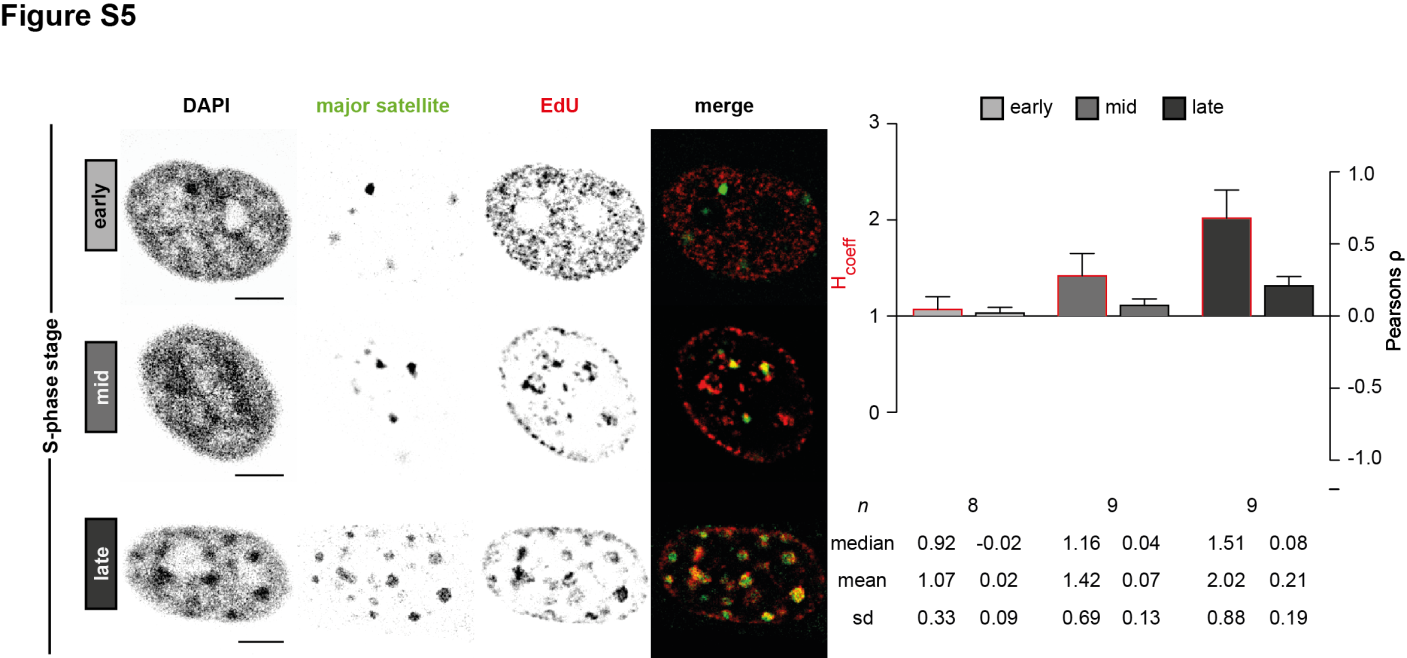


**Figure S5: Replication timing of murine major satellite DNA elements by FISH and S-phase sub-stages classification.** (left) Representative confocal and deconvolved micrographs of C2C12 cells depicting the DAPI, major satellite elements and EdU as inverted grey channels, at the three different S-phase substages. Merge is shown in pseudo-colors. Scale bar: 5 µm. (right) Colocalization analysis of FISH and EdU signal at the three different S-phase substages by H_coefficent_ and Pearson’s correlation coefficient as indicated. Error bars show the standard error of the mean. Data are from three independent experiments. *n*: combined total number of cells analyzed. sd: standard deviation.


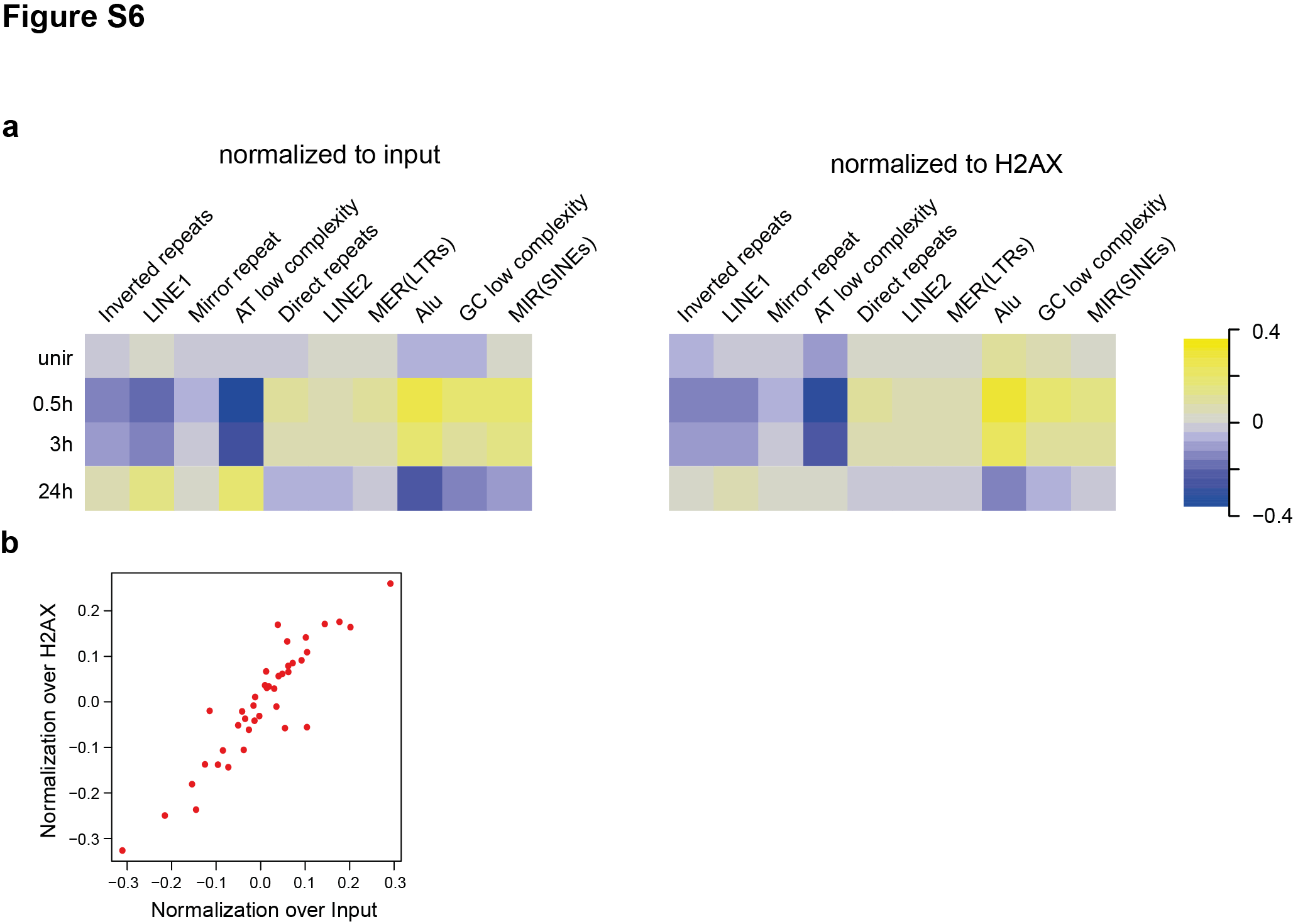


**Figure S6. Genome-wide correlation of DNA repetitive elements and histone γH2AX in HeLa cells.** (a) Spearman’s rho correlation matrices between DNA repetitive elements and γH2AX abundance before and after ionizing radiation. The matrices have been produced by normalizing the γH2AX ChIP sequencing abundance over the input (left) or the corresponding non-phosphorylated H2AX histone (right). Data are from >290,000 genomic intervals with P < 2.2 × 10^-16^. (b) Spearman’s rho correlation coefficients from data normalized over H2AX as a function of their matched coefficient normalized over the input. Normalization over H2AX or over input shows only minor differences (Pearson’s coefficient: 0.91).


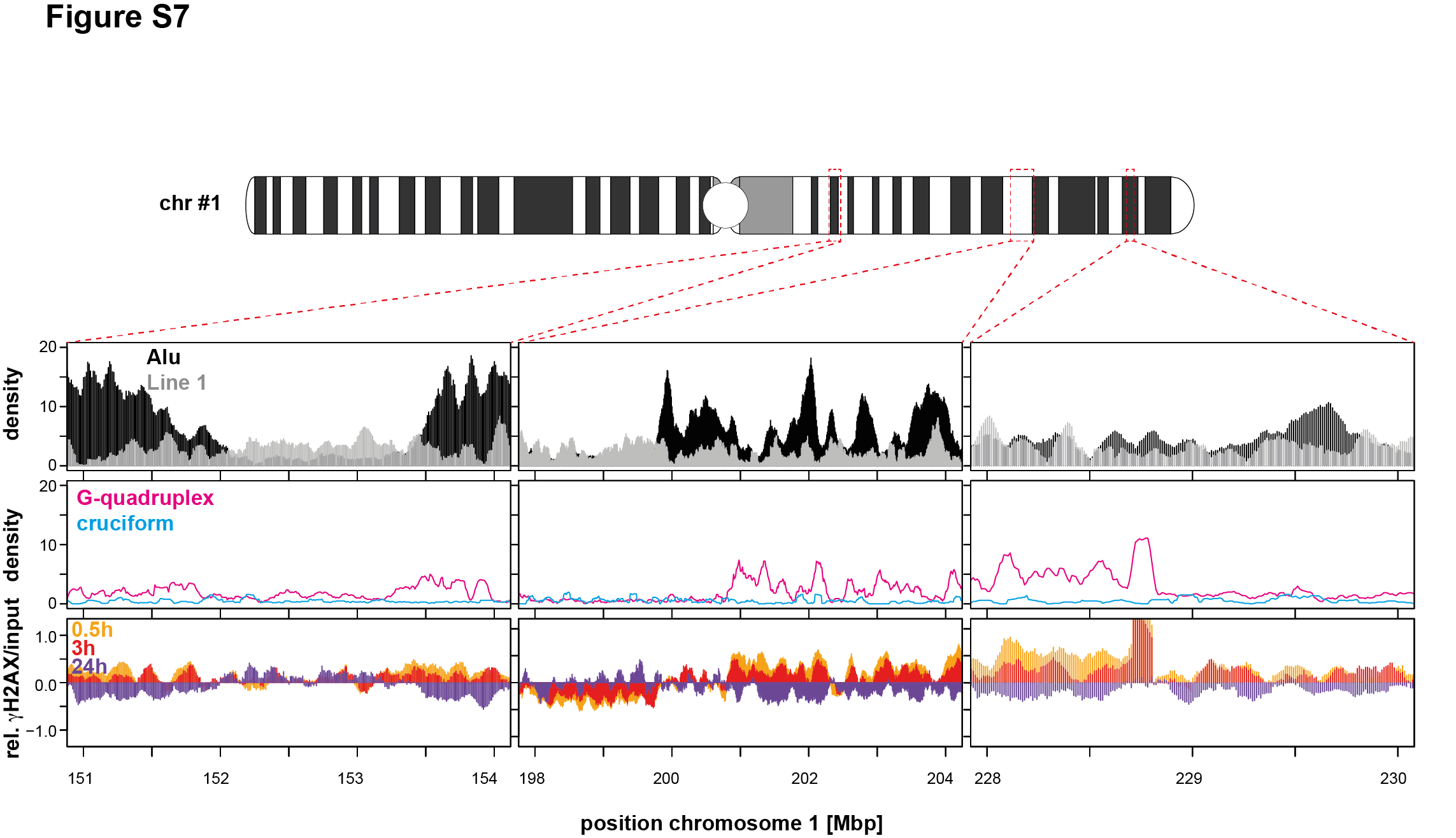


**Figure S7. Genomic repetitive and non-B DNA elements, and γH2AX histone distributions.** Exemplary genomic (top) Alu and LINE1, (mid) G-quadruplex and cruciform motifs and (bottom) γH2AX distributions in 10 kbp intervals from HeLa cells. For the latter, the histone modification abundance at 0.5, 3 and 24 hours post ionizing radiation is shown. For Alu and LINE1 elements as well as for G-quadruplex and cruciform motifs, the number of elements per interval is shown. All tracks were smoothed using a moving average of 10 intervals.

**
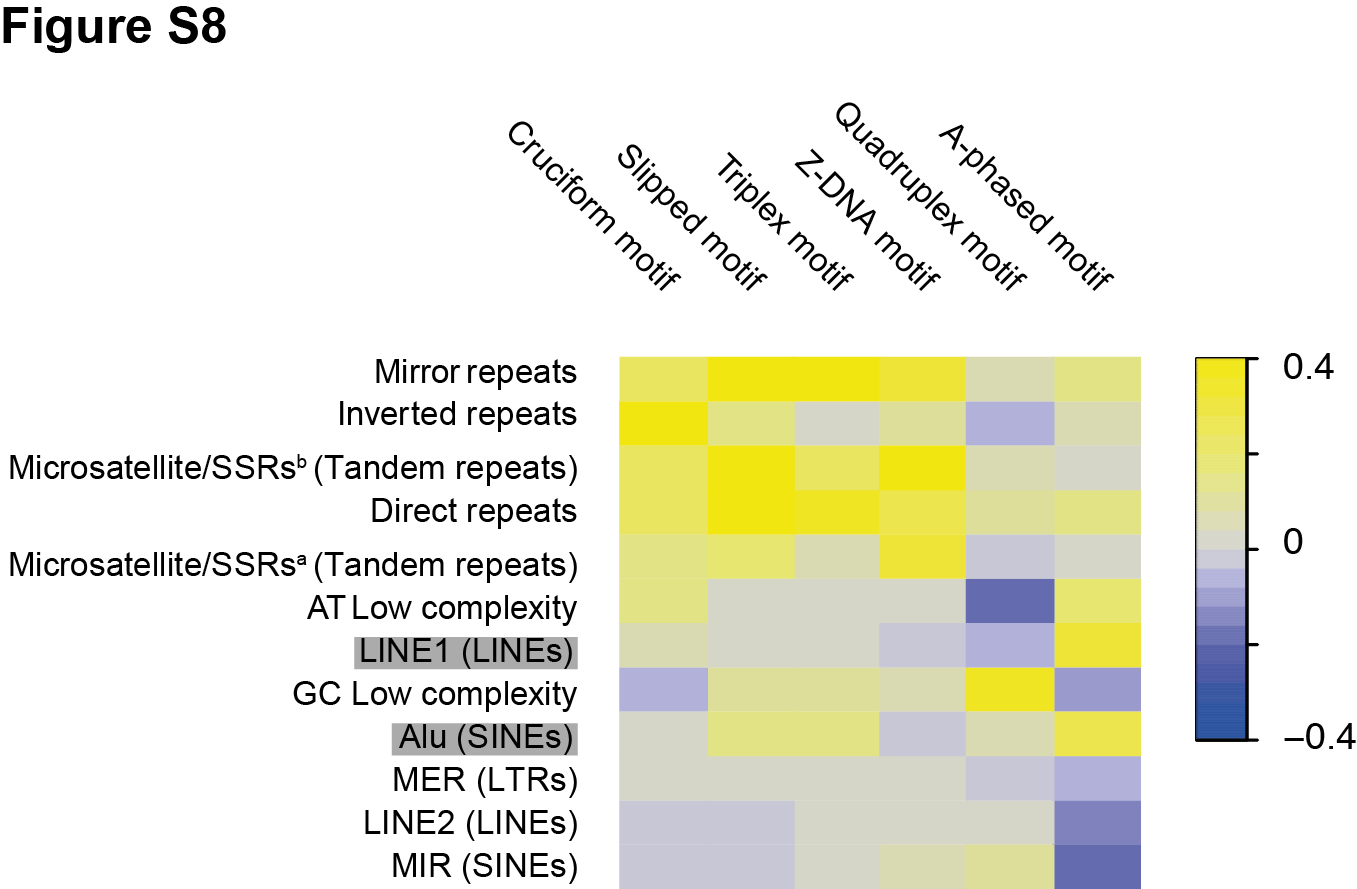
**

**Figure S8: Relation of repetitive DNA elements to non-B DNA elements.** Spearman’s rho correlation matrix. The number of each repetitive or non-B DNA element copies is computed in each 10 kb genomic interval. The correlation coefficient is then calculated. Data are from >290,000 genomic intervals. For each correlation, P < 2.2 × 10^-16^. Highlighted Alu and L1 repetitive elements are arbitrarily chosen to define chromatin compartments with opposing chromatin features, and are further investigated in FISH experiments.


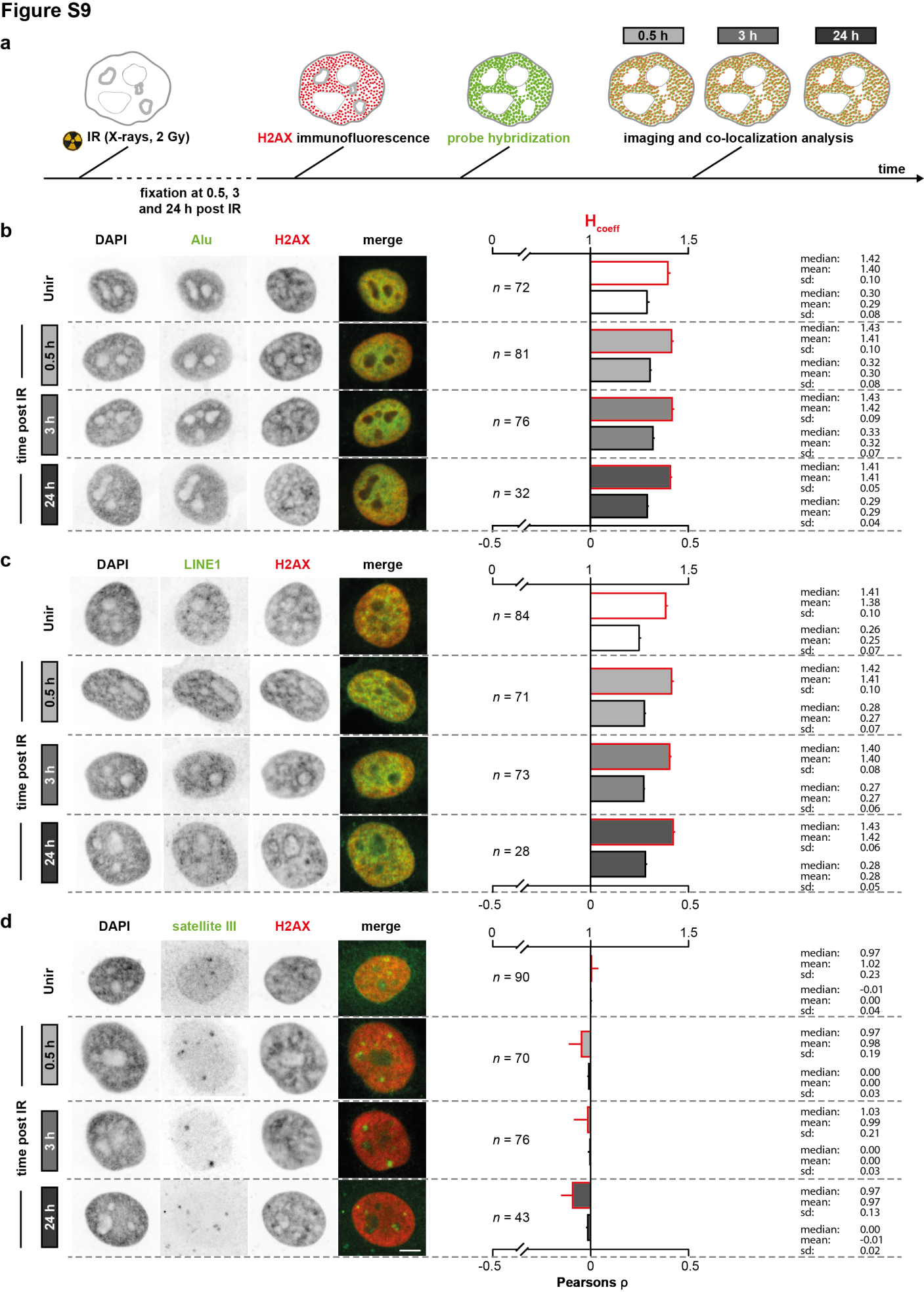


**Figure S9: Correlation of histone H2AX and repetitive DNA elements before and during the DDR by FISH.** (a) Schematics of the experiment. HeLa cells were sham-irradiated or irradiated with 2 Gy X-rays and incubated for 0.5, 3 and 24 hours. H2AX immunofluorescence and probe hybridization were performed before confocal imaging. (b) (left) Representative confocal micrographs of HeLa cells depicting the DAPI, Alu elements and H2AX inverted grey channels, at the indicated times. Merge is shown in pseudo-colors. Scale bar: 5 µm. (right) Colocalization analysis of FISH and H2AX signals via H_coefficent_ and Pearson’s correlation coefficient as indicated. Error bars show the standard error of the mean. Data are from three independent experiments. *n*: combined total number of cells analyzed. sd: standard deviation. (c) and (d) represent the same as in (b) for L1 and satellite III, respectively.


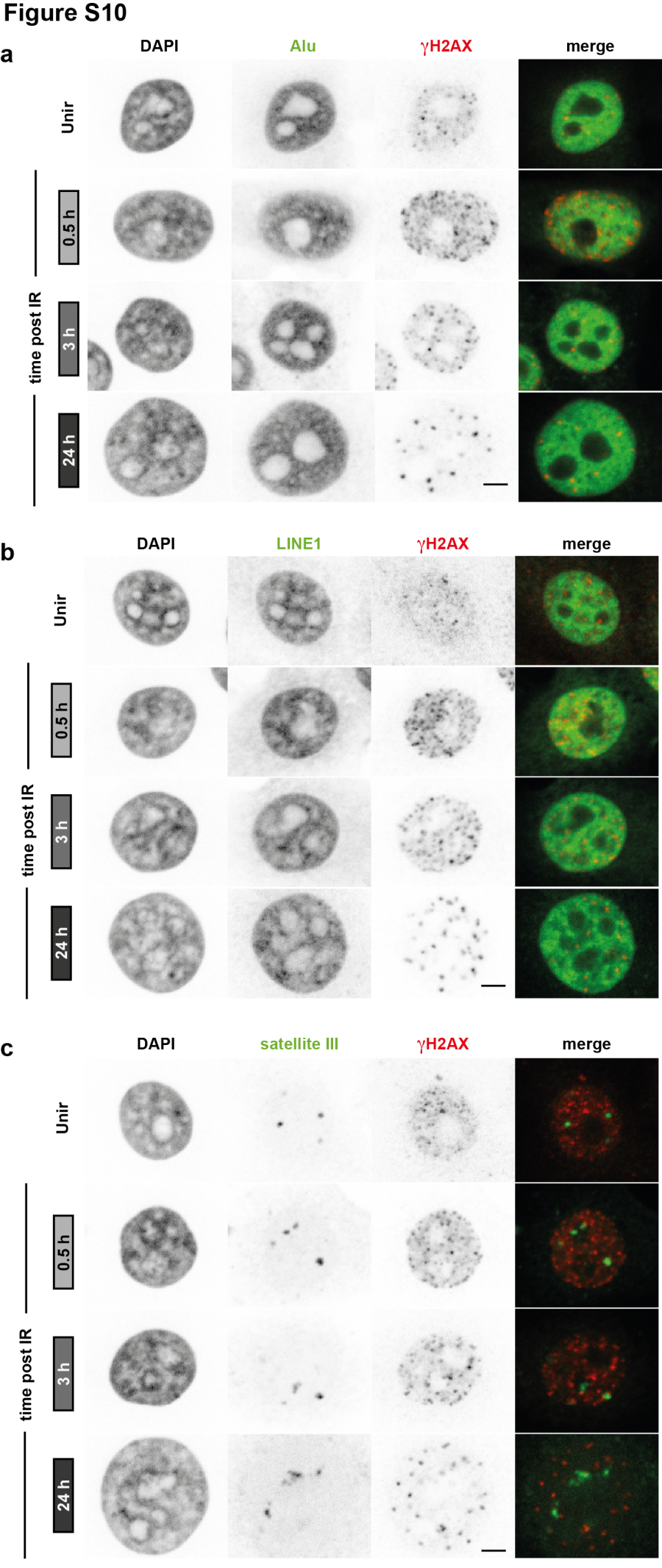


**Figure S10. Complete DNA repair kinetics of repetitive DNA elements analyzed by FISH.** (a) Representative confocal images of HeLa cells depicting the DAPI, Alu elements and γH2AX as inverted grey channels, before irradiation (top) and at the three time-points post IR. Merge is also shown in pseudo-colors. Scale bar: 5 µm. (b) and (c) represent the same as in (a) for L1 and satellite III, respectively.


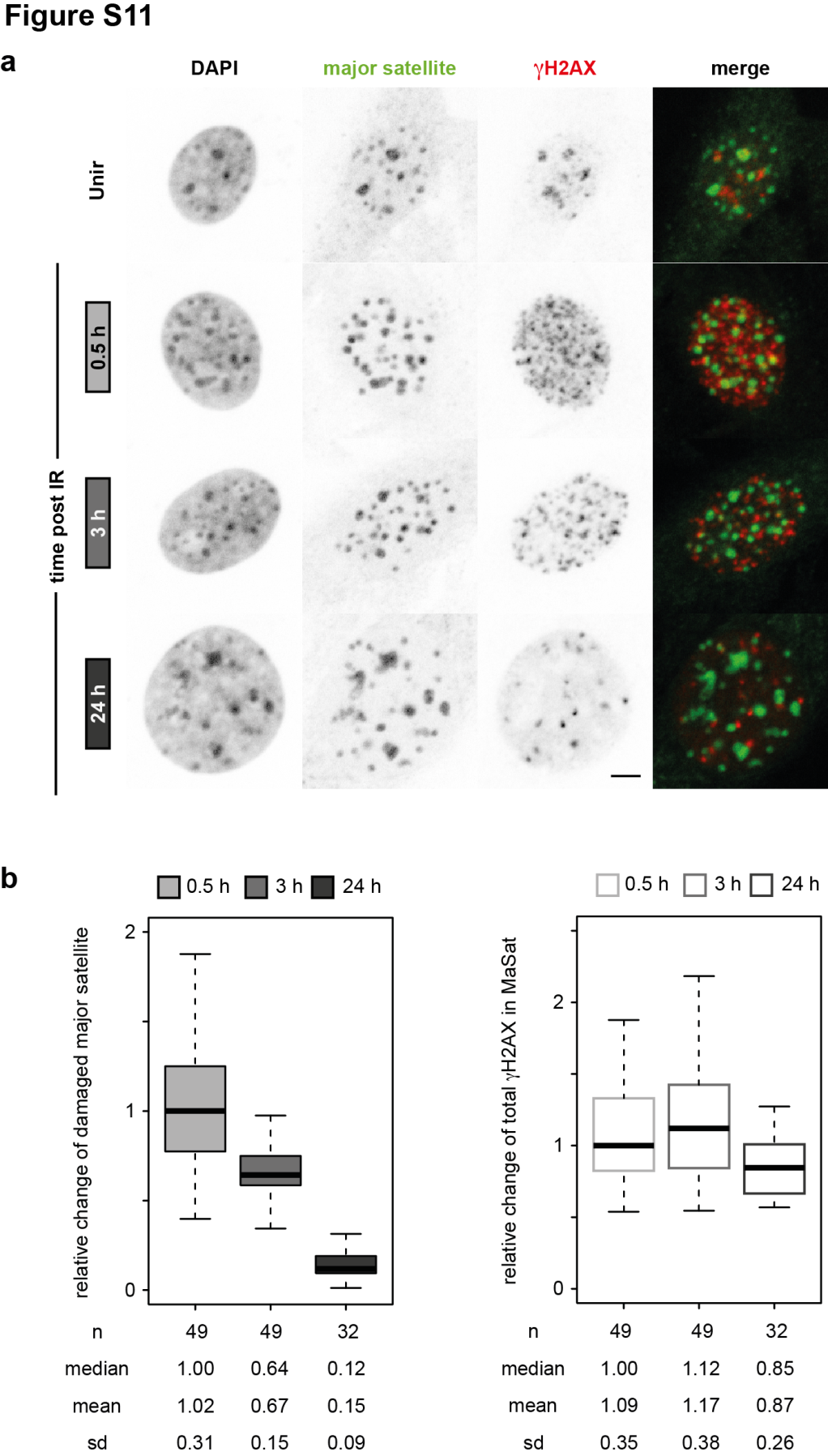


**Figure S11: DNA repair kinetics of murine major satellite DNA elements analyzed by FISH.** (a) Representative confocal images of C2C12 mouse myoblast cells depicting the DAPI, major satellite elements and γH2AX as inverted grey channels, before and at the three time-points post IR. Merge is shown in pseudo-colors. Scale bar: 5 µm. (b) (left) Relative change of major satellite fraction in γH2AX foci. (right) Relative change of γH2AX intensity in segmented major satellite regions. Data are normalized to the median of the 0.5 h time-point. Boxes represent median, 2^nd^ and 3^rd^ quartile. Whiskers are three times the interquartile distance. Data are from three independent experiments. *n*: combined total number of cells analyzed. sd: standard deviation.
